# Systemic Supplementation of Collagen VI by Neonatal Transplantation of iPSC-Derived MSCs Improves Histological Phenotype and Function of Col6-Deficient Model Mice

**DOI:** 10.3389/fcell.2021.790341

**Published:** 2021-11-23

**Authors:** Aya Harada, Megumi Goto, Atsuya Kato, Nana Takenaka-Ninagawa, Akito Tanaka, Satoru Noguchi, Makoto Ikeya, Hidetoshi Sakurai

**Affiliations:** ^1^ Department of Clinical Application, Center for iPS Cell Research and Application (CiRA), Kyoto University, Kyoto, Japan; ^2^ Department of Neuromuscular Research, National Institute of Neuroscience, National Center of Neurology and Psychiatry, Tokyo, Japan

**Keywords:** iPS cell, mesenchymal stromal cells, COL6-related myopathy, systemic cell transplantation, ullrich congenital muscular dystrophy (UCMD)

## Abstract

Collagen VI is distributed in the interstitium and is secreted mainly by mesenchymal stromal cells (MSCs) in skeletal muscle. Mutations in *COL6A1-3* genes cause a spectrum of COL6-related myopathies. In this study, we performed a systemic transplantation study of human-induced pluripotent stem cell (iPSC)-derived MSCs (iMSCs) into neonatal immunodeficient COL6-related myopathy model (*Col6a1* ^*KO*^/NSG) mice to validate the therapeutic potential. Engraftment of the donor cells and the resulting rescued collagen VI were observed at the quadriceps and diaphragm after intraperitoneal iMSC transplantation. Transplanted mice showed improvement in pathophysiological characteristics compared with untreated *Col6a1* ^*KO*^/NSG mice. In detail, higher muscle regeneration in the transplanted mice resulted in increased muscle weight and enlarged myofibers. Eight-week-old mice showed increased muscle force and performed better in the grip and rotarod tests. Overall, these findings support the concept that systemic iMSC transplantation can be a therapeutic option for COL6-related myopathies.

## Introduction

Ullrich congenital muscular dystrophy (UCMD), which is regarded as the severe end of COL6-related myopathy, is a life-threatening muscular and connective tissue disorder, characterized by early-onset muscle weakness with multiple joint contractures and distal joint hyperlaxity (Bönnemann, 2011). The onset has been reported to be 12 months on average (Nadeau et al., 2009); nevertheless, multiple joint contractures at birth are evident in early severe cases (Yonekawa and Nishino, 2015). Reduced fetal movement leading to prenatal diagnosis was also identified (Hata et al., 2018). Typically, ambulation can be achieved only for a limited period, and respiratory muscle atrophy and scoliosis cause impaired respiratory function (Yonekawa et al., 2013). Mutations in *COL6A1*, *COL6A2,* and *COL6A3* genes, identified with both recessive and mostly *de novo* dominant inheritance patterns, have different effects on collagen VI synthesis, assembly, secretion, and function, generating a phenotypically variable spectrum of COL6-related myopathies (Lamandé and Bateman, 2018).

Collagen VI null mice have contributed to our understanding of the pathomechanisms of COL6-related myopathies (Bonaldo et al., 1998; Noguchi et al., 2017). *Col6a1* ^*−/−*^ mice were produced by inserting a neomycin resistance cassette into the second exon of the *Col6a1* gene, which truncated the mRNA transcript. Without α1 (VI) chain, triple helical collagen VI molecules are not expressed in the mutant mice (Bonaldo et al., 1998; Lamandé et al., 1998). On the contrary, *Col6a1* ^*GT/GT*^ mice were generated by knocking in a point mutation in exon 9 in *Col6a1* gene (Noguchi et al., 2017). The resulting miss-splicing after exon 9 causes a marked reduction in normal *Col6a1* mRNA expression and a premature stop codon, resulting in *Col6a1* knockout and the mice having no collagen VI protein in all tissues (Noguchi et al., 2017). Therefore, we refer to *Col6a1* ^*GT/GT*^ mice as *Col6a1* ^*KO*^ mice in this manuscript. Both conventional *Col6a1* ^*−/−*^ mice and *Col6a1* ^*KO*^ mice are phenotypically similar, but *Col6a1* ^*KO*^ mice have a smaller body weight (BW) than WT mice (Bonaldo et al., 1998; Noguchi et al., 2017). Furthermore, although histological phenotypes, such as variations in myofiber size and an increased number of myofibers with central nuclei, are observed in both *Col6a1*-mutated mouse types, a more quantitative analysis is available for *Col6a1* ^*KO*^ mice. Researchers have confirmed that the number of myofibers of very small diameter is increased and the total number of myofibers in the tibialis anterior (TA) muscles starts to be reduced from postnatal day 15 onward, indicating a defect in muscle growth and signaling during development in *Col6a1* ^*KO*^ mice (Noguchi et al., 2017). Since consistent findings were found in muscle biopsies from UCMD patients (Higuchi et al., 2003), *Col6a1* ^*KO*^ mice are considered UCMD model mice. In contrast, the limited quantitative histology data of *Col6a1* ^*−/−*^ mice have made it difficult to define *Col6a1* ^*−/−*^mice as UCMD model mice.

The first clue of a therapeutic approach for COL6-related myopathies came from evidence that mitochondrial dysfunction triggers the apoptosis of myofibers and medication (Irwin et al., 2003; Bernardi and Bonaldo, 2008) and that autophagy is impaired in *Col6a1* ^*-/-*^ mice as well as in UCMD patients (Grumati et al., 2010; Bernardi and Bonaldo, 2013). Cyclosporine A and cyclophilin D both decreased the number of apoptotic events of myofibers by increasing mitochondrial tolerance for depolarized stimulation (Bernardi and Bonaldo, 2008; Tiepolo et al., 2009; Merlini et al., 20112011; Gattazzo et al., 2014), and forced activation of autophagy by a low protein diet benefited the muscle homeostasis of autophagic markers (Castagnaro et al., 2016). Alternatively, allele-specific knockdown strategies demonstrated the restored expression of collagen VI in the fibroblasts of UCMD patients (Bolduc et al., 2014; Noguchi et al., 2014; Marrosu et al., 2017; Bolduc et al., 2019). However, the above approaches are limited to normalizing the autophagic flux, which is not sufficient for improving the muscle function adequately (Merlini et al., 20112011) or unsuitable for long-term use (Castagnaro et al., 2016). Further, gene therapies target only specific mutations and cannot be applied to most patients with different pathogenic variants (Lampe AK et al., 2004). On the contrary, the therapeutic potential of mesenchymal cell transplantation has been recognized since the source of collagen VI in skeletal muscle is interstitial fibroblasts, not myogenic cells (Zou et al., 2008). The local intramuscular injection of adipose-derived mesenchymal stem cells (ADSCs) from neonatal skin resulted in the long-term and continuous secretion of collagen VI in *Col6a1* ^*−/−*^ *Rag1* ^*−/−*^mice, but neither the histological nor functional effects were investigated (Alexeev et al., 2014).

iMSCs are mesenchymal stromal cells (MSCs) derived from human induced pluripotent stem cells (iPSCs) (Fukuta et al., 2014; Matsumoto et al., 2015; Chijimatsu et al., 20172017) and share comparable gene and protein expression levels of collagen VI with primary MSCs from adult skeletal muscles (Takenaka-Ninagawa et al., 2021). When injected locally into the TA muscles of *Col6a1* ^*KO*^/NSG mice, iMSCs secrete collagen VI for a long time to enhance muscle regeneration at the lesion where collagen VI was restored (Takenaka-Ninagawa et al., 2021). Therefore, we investigated whether systemic iMSC transplantation can potentially restore collagen VI expression throughout the skeletal muscles of the whole body in *Col6a1* ^*KO*^/NSG mice and ameliorate the pathophysiology. We also expected that neonatal intervention would have a physiological influence on muscle development from an earlier period and produce better effects with a smaller number of donor cells.

Accordingly, this study aimed to prove the therapeutic effects of systemic iMSC transplantation in *Col6a1* ^*KO*^/NSG mice. We demonstrated that the intraperitoneal (i.p.) injection of iMSCs contributed to the expression of collagen VI among skeletal muscles and increased the size and number of myofibers by upregulating muscle regeneration. The number of abnormal mitochondria in the treated *Col6a1* ^*KO*^/NSG mice was decreased, suggesting iMSC transplantation may provide some positive environmental changes to *Col6a1*-null muscles. Functional improvements by the transplantation were also achieved.

## Materials and Methods

### Mice

UCMD model mice were generated as previously described (Noguchi et al., 2017). In order to distinguish these mice from conventional *Col6a1* ^*−/−*^ mice, we call them *Col6a1* ^*KO*^ mice. *Col6a1* ^*KO*^ mice were backcrossed to achieve the NSG (NOD acid gamma) background. After establishing the *Col6a1* ^*HETERO*^/NSG mice line, we crossed the mice again to produce *Col6a1* ^*KO*^/NSG mice. Because *Col6a1* ^*KO*^/NSG mice are hard to conceive, the offspring were maintained by *in vitro* fertilization (IVF) with ICR surrogate mothers. We used 3–8 generations of the *Col6a1* ^*KO*^/NSG strain. The mice were kept under a 12/12 h light-dark cycle with easy and comfortable access to food and water. The pups were weaned at 3 weeks of age. The genotype of the offspring confirmed no contamination of *Col6a1* ^*HETERO*^/NSG mice. WT/NSG mice (NOD.Cg-*Prkdc* ^*scid*^ *Il2rg* ^*tm1Wjl*^/Szj) were purchased from Charles River Laboratory and kept under the same environment as *Col6a1* ^*KO*^/NSG mice.

### Cells

The differentiation of 201B7 iPSCs to iMSCs via iPSC-derived neural crest cells (iNCCs) was performed as previously published (Fukuta et al., 2014). Luciferase-expressing piggyBac vector (pPV-EF1a-Luc2-iP-A) (Xu et al., 2019) and piggyBac transposase plasmids (pHL-EF1a-hcPBase-A) were introduced into the iNCCs by electroporation with a NEPA21 electroporator (Nepa Gene, Co., Ltd., Chiba, Japan), and the transfected cells were selected with 0.5% puromycin over 7 days. Luciferase-expressing iNCCs were maintained by passaging after 90% confluency or kept frozen in cryopreservation liquid (Bambanker, Nippon Genetics Co., Ltd., CS-02-001) as a stock. The iNCCs were induced to iMSCs just before use. Only passage 5 or 6 iMSCs were used for the transplantation.

### Transplantation

Only male pups were used for analysis throughout the study. For neonatal i. p. transplantation, 5 × 10^6^ iMSCs in 20 μl phosphate-buffered saline (PBS) were injected intraperitoneally using a 30G insulin syringe (BD, No. 326668). All the mice analyzed at 8 weeks had a boost i. p. transplantation at 4 weeks, in which 5 × 10^6^ iMSCs in 100 μl PBS were injected using a 23G 1 ml syringe (Terumo, SS-01T2613).

### *In Vivo* Imaging System

Mice were anesthetized with isoflurane, and the luminescence was imaged with an IVIS imaging system (Parkin Elmer) 10 min after the administration of 150 mg/kg d-luciferin substrate (Summit Pharmaceutical International Corporation, XLF-1) in PBS. The luminescence intensity was measured, and the region of interest (ROI) was set to cover all the signals.

### RT-PCR

cDNA used for *luciferase* detection was treated as follows. After the removal of DNA contamination with DNase I (Invitrogen, 18068-015), 1 μg mRNA was reverse transcribed with SuperScript III using a random hexamer as instructed (Invitrogen, 18080-051). The PCR products were electrophoresed on a 1% agarose gel for 30 min to detect amplified fragments of luciferase and imaged under a FAS-IV gel imaging device (Nippon Genetics, Co., Ltd.). Positive control samples were dissected from a teratoma that had been formed 8 weeks after the subcutaneous injection of luciferase-integrated iPSCs into the TA muscle of WT/NSG mice. Negative control samples were extracted from the TA muscle of *Col6a1* ^*KO*^/NSG mice.

### Tissue Sectioning

Mice were euthanized by exposure to gradually increasing concentrations of carbon dioxide. Dissected TA and quadriceps were stuck onto the pedestal by tragacanth gum (Wako, 206-02242) on a cork and frozen by dipping into 2-methylbutane (Wako, 166-00615) for 45–60 s at melting point temperature (−156°C). Freshly isolated diaphragms were embedded in OCT compound (Sakura Finetek, 4583, USA) and directly frozen within 2-methylbutane. Frozen muscles were sliced at 12-μm thickness with a Cryostat (Leica, CM1850) and attached to the slide. The sample on the slide was kept at −80°C for the following immunostaining.

### Immunohistochemistry

The frozen sample on the slide was fixed in 4% paraformaldehyde for 20 min and washed twice with PBS. After 1-h incubation with Blocking one (Nacalai tesque, 03953-95), the primary antibody in immunoreaction enhancer solution (Can get signal, TOYOBO, NKB-601) was applied to the sample overnight at 4°C. The sample was washed with 0.1% Triton-X in PBS three times for 10 min each. The secondary antibody was applied for 1 h at room temperature, and the sample was washed three times. Images were obtained under a confocal microscope (Zeiss, LSM710). The primary and secondary antibodies used for the immunostaining and the dilution ratios are described in Supplementary Table S1.

### Cell Apoptosis Assay

TUNEL positive cells were stained with an Apoptag® plus peroxidase *in situ* apoptosis detection kit (Millipore, S7101) following the manufacturer’s instructions. After the TUNEL staining, an additional course of immunostaining for MYH3 was performed as described above. The fixation and blocking steps were skipped for the second staining.

### Functional Analysis

We planned three different functional tests with the same mice. All tests were conducted within 1 week in the same order: muscle force first, grip test second, and rotarod test third. Non-transplanted and transplanted mice were the siblings of the same surrogate ICR mothers and were randomly selected into either group. They grew up together until weaning at 3 weeks. After weaning, the mice were divided into same-sized cages with five mice each.

#### Functional Analysis of Maximum Isometric Torque

A custom-made mouse ankle joint motor function analysis system (Bio Research Center, Nagoya, Japan) was used as previously described (Itoh et al., 2017). Briefly, the mice were anesthetized with isoflurane inhalation, and the plantar was attached to the pressure sensor. Two electrodes were attached to the shaved hind limb with viscous electrical conductive gel (CR-S; Sekisui Plastics, Osaka, Japan) in between. One was fixed to the myotendinous junction and the other was fixed 5 mm above it with adhesive tape. Electrical stimulation was applied to the skin surface of the triceps surae muscle using an electric stimulator (SEN-3301; Nihon Kohden, Tokyo, Japan) to induce muscle contraction. Isometric plantarflexion torque was calculated from the pressure applied to the sensor and the distance from the ankle joint to the sensor. The measurement was performed twice for each foot, and the average value was adopted.

#### Grip Strength Test

All measurements were blindly taken by one experienced examiner (M.G.). After being calmed on the examiner’s hand, the mice were placed on wire mesh equipped with a traction meter (no. BS-TM-RM, Brain Science idea, Osaka, Japan) by gripping with all four extremities. The examiner pulled the tail, and the maximum value of the grip strength was measured three times. The average of the three tests was counted as the score of an individual mouse.

#### Rotarod Test

The test run was performed once a day on a rotarod apparatus (Ugo Basile, Cat No.47600). After 2 runs as mock exams, the duration of the running time was recorded for 3 days. The rotation speed was started at 4 rpm, increased to 50 rpm in 300 s, and kept at 50 rpm for another 300 s. The maximum running time was 600 s. The average of three runs was counted as the score of an individual mouse.

### Statistics

Numerical data with three groups were analyzed with a one way-ANOVA followed by the Tukey-Kramer method. The student’s t-test was performed between two independent groups. *p < 0.05, **p < 0.01, and ***p < 0.001 are labeled in the figures. ^†^ was used when *p*-values were <0.1, indicating that the two groups tended to have a difference, but it was not statistically different.

### Study Approval

All animal experiments were performed in accordance with the guidelines of the animal experiment committee and recombinant DNA experiment committee at Kyoto University. The study design was reviewed and accepted by the animal experiment committee in advance of conducting all the experiments (No.17-95-2).

## Results

### iMSCs Reached Skeletal Muscle and Secreted Collagen VI Locally After Intraperitoneal Injection

**FIGURE 1:**
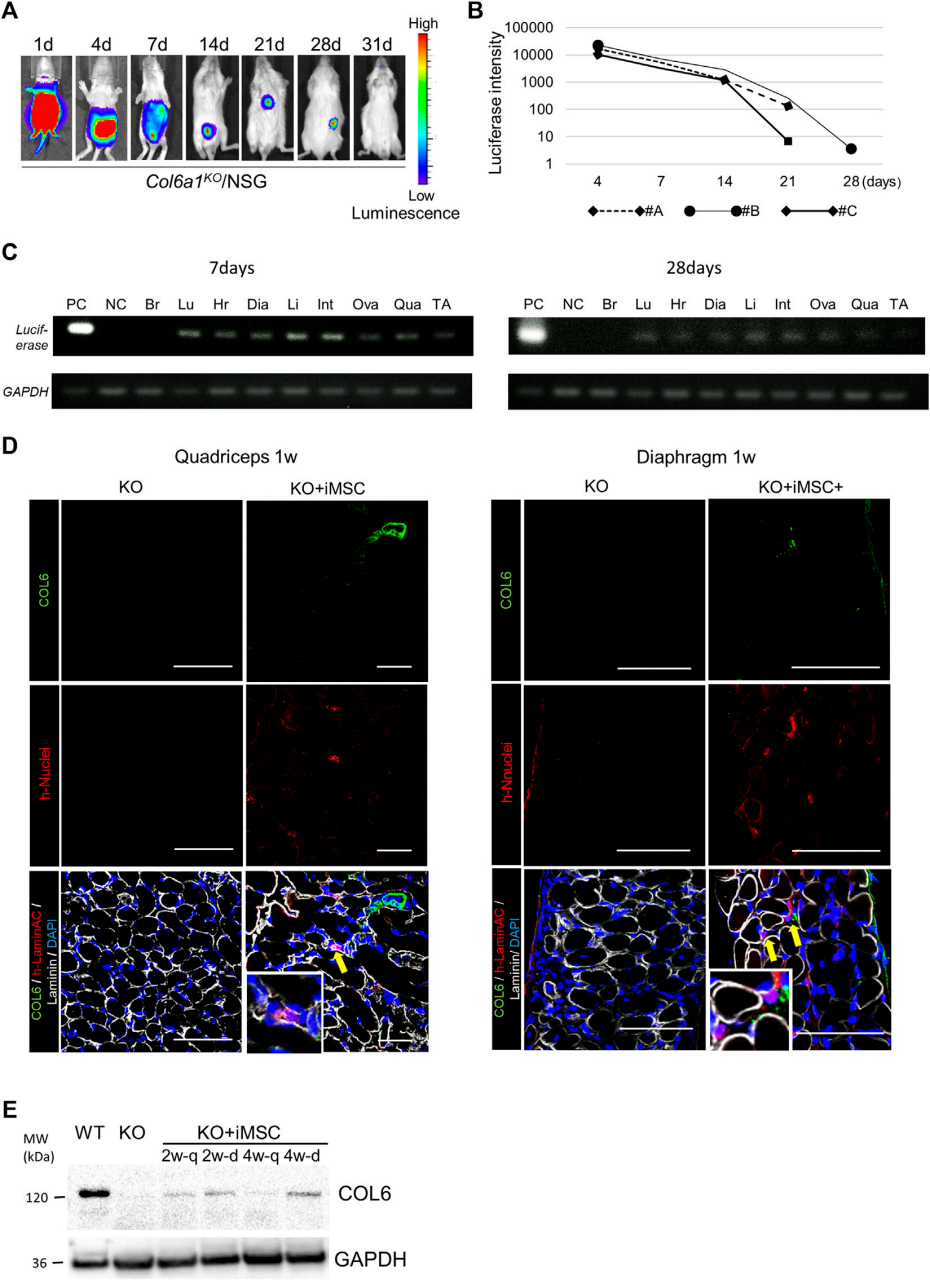
Tracking of donor cells after intraperitoneal transplantation. **(A)** *In vivo* imaging of the donor cells. The signal from the donor cells of #B in Figure 1B is shown. The signal disappeared after 4 weeks. **(B)** Time course of the luciferase signal intensity of three mice. **(C)** *Luciferase* detection by PCR 7 days (top) and 28 days (bottom) after the transplantation. 0.8 μg mRNA from each organ was extracted, and the donor cells in each organ were detected by luciferase and GAPDH primers. The signal was detected in the lung (Lu), heart (Hr), diaphragm (Dia), liver (Li), intestine (Int), uterus and ovary (Ova), quadriceps (Qua), and tibialis anterior muscle (TA), but not the brain (Br). The positive control (PC) was extracted from a teratoma dissected 8 weeks after the subcutaneous injection of iPSCs with luciferase integration. NC: negative control. **(D)** Immunofluorescence staining of the quadriceps (left) and diaphragm (right) 7 days after iMSC intraperitoneal transplantation. Donor cells were positive for human-nuclei (yellow arrows). Magnifications of the human-nuclei positive cells are also shown. Scale bars, 50 μm. COL6: collagen VI. **(E)** Western blotting for the detection of collagen VI. Proteins from the quadriceps and diaphragm of *Col6a1* ^*KO*^/NSG mice 2 and 4 weeks after neonatal transplantation were extracted. PC was a diluted sample (1:10) extracted from the quadriceps of WT/NSG mice at 4 weeks. NC was a sample from the quadriceps of *Col6a1* ^*KO*^/NSG mice. All blots were derived from the same experiment and processed in parallel.

First, we confirmed that the donor cells were positive for CD73, CD44, and CD105 and negative for CD45 and HLA-DR (Supplementary Figure S1A). The expression of collagen VI was also confirmed (Supplementary Figure S1B). Next, we evaluated the potential of human iMSCs to deliver collagen VI to the skeletal muscles in *Col6a1* ^*KO*^/NSG mice after neonatal i. p. injection. Two days after birth, 5 × 10^6^ luciferase-expressing iMSCs were transplanted to the peritoneal cavity, and their distribution was observed with an IVIS imaging system. The transplanted cells were widely delivered including to the lower and upper extremities after 24 h (Figure 1A). The total intensity of the luciferase signal decreased with time but was detectable for 28 days (Figure 1B). iMSCs were seen in major organs except for the brain based on *luciferase* transcript expressions at 7 days after the transplantation (Figure 1C). Fewer iMSCs were detected at 28 days.

Histological analysis revealed that the iMSCs had reached the interstitial space of the quadriceps and diaphragm 1 week after the transplantation (Figure 1D). The expression of restored collagen VI was also confirmed by Western blotting in the quadriceps and diaphragm after 2 weeks (Figure 1E). Collagen VI expression in the tissues suggested that iMSCs migrated to the skeletal muscle and secreted collagen VI locally.

To prove that iMSCs can migrate throughout the body via blood vessels, we performed intravenous (i.v.) transplantation from the facial vein 1 day after birth. The transplanted cells distributed to the whole body including the quadriceps and diaphragm, where they secreted collagen VI (Supplementary Figures S2A,B). Collagen VI secretion was sustained for more than 28 days in the limb muscles (Supplementary Figure S2C). Although the i.v. transplantation had comparable results with the i. p. transplantation, we chose to perform further experiments with i. p. transplantation because i. v. transplantation was technically difficult. Overall, the above findings indicate that transplanted iMSCs can deliver molecules into organs including skeletal muscle, suggesting a potential for treating systemic diseases.

### iMSC Transplantation Positively Affected the Phenotype of *Col6a1* ^*KO*^/NSG Mice

Next, we searched for indicators to evaluate the effects of the transplantation on skeletal muscle. At 4 weeks, there was no positive effect on muscle weight after the transplantation (Supplementary Figure S3A), although the engraftment of donor cells and expression of collagen VI in the quadriceps and diaphragm were confirmed (Supplementary Figure S3B). A histogram of the diameters of the quadriceps revealed that the myofiber size was smaller in *Col6a1* ^*KO*^/NSG mice than in wild type (WT)/NSG mice, and its distribution shifted and became larger in the transplanted mice (Supplementary Figure S3C). The percentage of small myofibers and large myofibers decreased and increased, respectively (Supplementary Figure S3D), and the average size of the myofibers was increased after the transplantation (Supplementary Figure S3E). The area of the myofibers tended to increase (*p* = 0.064) (Supplementary Figures S3F,G).

**FIGURE 2:**
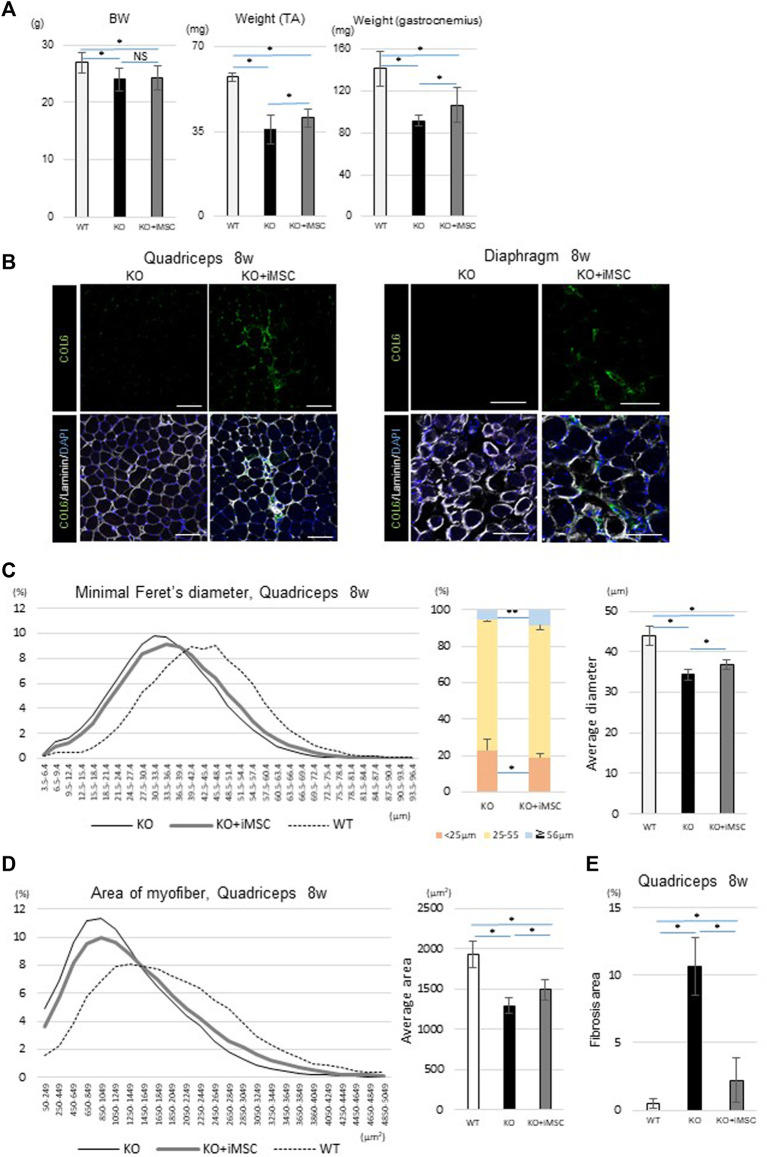
Phenotypes at 8 weeks. **(A)** Body weight (BW) and muscle weight of each group. For BW: WT (*n* = 15), KO (*n* = 66), and KO + iMSC (*n* = 30). For raw weight of the TA (middle) and gastrocnemius muscles (right): WT (*n* = 6), KO (*n* = 9), and KO + iMSC (*n* = 13). (Tukey’s test). **(B)** Immunofluorescence staining of the quadriceps (left) and diaphragm (right) in non-transplanted (KO) and transplanted mice (KO + iMSC). The transplanted mice showed collagen VI expression in the interstitial space of the muscles. Scale bars, 50 μm. **(C,D)** Histological analysis of myofibers in the quadriceps. **(C)**: Diameter of the short axis (minimal Feret’s diameter). Left: histogram of the diameter; middle: frequency of different diameter groups; right: average diameter. **(D)**: Area of the myofibers. Left: histogram of the myofiber size; right: average size. WT (*n* = 7), KO (*n* = 9), and KO + iMSC (*n* = 13). **(E)** Fibrotic area in the quadriceps calculated by Sirius red staining. WT (*n* = 4), KO (*n* = 5), and KO + iMSC (*n* = 5). (Tukey’s test). All error bars indicate ± SD.

When a boost i. p. injection of 5 × 10^6^ iMSCs was added at 4 weeks, the therapeutic effects of the transplantation were more clearly demonstrated at 8 weeks than at 4 weeks. Donor cells after the second i. p. transplantation were dominantly observed in the interstitium, suggesting that the transplanted cells were extravasated from the blood vessels to the quadriceps (Supplemental Figure S4A). An IVIS study confirmed similar iMSC dynamics with neonatal transplantation, with the period of the luciferase signal ranging from 2 to 4 weeks (Supplementary Figure S4B). Muscle weight at 8 weeks was increased in the transplanted mice compared to the non-transplanted mice, although BW was not significantly changed (Figure 2A). Donor cells were much less detected than at 4 weeks, but collagen VI expression had dispersed and remained in the quadriceps and diaphragm of the transplanted mice (Figure 2B). Histological analysis of the quadriceps showed the diameter and area of myofibers were increased with the transplantation (Figures 2C,D).

Fibrosis in skeletal muscle is abundant in adult *Col6a1* ^*KO*^ mice (Noguchi et al., 2017). On the other hand, one of the concerns of injecting iMSCs is that iMSCs may induce fibrosis depending on their microenvironment because mesenchymal progenitor cells in skeletal muscle can be a source of both fat accumulation and fibrosis in diseased mice (Uezumi et al., 2011). To understand the impact of the iMSC transplantation on fibrosis formation, we performed several studies. Sirius red staining showed that the fibrotic change in the quadriceps was more prominent in non-transplanted *Col6a1* ^*KO*^/NSG mice than in WT/NSG mice but was ameliorated with the transplantation (Supplementary Figure S5A). Quantitative analysis of the fibrotic area also showed suppressed fibrosis in the transplanted mice (Figure 2E). The gene expression of *Postn*, a matricellular protein that contributes to fibrosis (Kanaoka et al., 2018), was suppressed in the diaphragm of transplanted mice (Supplementary Figure S5B), and reduced Periostin expression in the transplanted mice was confirmed by immunostaining (Supplementary Figure S5C).

In sum, iMSC-transplanted mice showed an increased myofiber size at 4 weeks. When the boost transplantation was added at 4 weeks, the muscle weight and myofiber size were increased at 8 weeks without any enhancement of fibrosis.

### Quantification of Rescued Collagen VI

The expression of collagen VI was measured by Western blotting and immunofluorescence staining in the quadriceps and diaphragm. The expression level of collagen VI in the Western blotting was detected in all samples at 4 weeks, but undetectable in one of the four samples at 8 weeks (Supplementary Figure S6A). The average expression level in the quadriceps sample of the transplanted mice was 0.48% at 4 weeks and 0.24% at 8 weeks that of WT mice at 4 weeks. In the diaphragm, the average percentages were 1.2 and 0.88%, respectively (Supplementary Figure S6B).

The collagen VI-rescued area based on immunofluorescence staining was also calculated (Supplementary Figures S7A, S8A). The collagen VI expression gradually increased after neonatal transplantation, reaching a peak at around 4–5 weeks, and decreased thereafter in the quadriceps even with boost transplantation at 4 weeks (Supplementary Figure S7B). The collagen VI-restored area at 4 weeks in the quadriceps was 5.87%, which is equivalent to 16.3% of the collagen VI positive areas in 4-week-old WT/NSG mice (Supplementary Figures S7B,C). The average collagen VI-rescued area at 8 weeks in the quadriceps was 3.45% (equivalent to 11.0% in 8-week-old WT/NSG mice) after neonatal and boost iMSC transplantation at 4 weeks, but 1.03% (equivalent to 3.31% in 8-week-old WT/NSG mice) when only neonatal transplantation was performed (Supplementary Figures 7B,C). Boost transplantation at 4 weeks slowed the rate at which collagen VI degraded, but it did not maintain the collagen VI-rescued area at 4 weeks. The donor cells in the quadriceps decreased with time and were rarely detected after 8 weeks (Supplementary Figure S7d).

As for the diaphragm, the average collagen VI-rescued area was 5.7% at 4 weeks (equivalent to 22.2% in 4-week-old WT/NSG mice) and 3.6% at 8 weeks (10.4% in 8-week-old WT/NSG mice) (Supplementary Figures 8B,C). The number of donor cells in the diaphragm showed a similar pattern as in the quadriceps and decreased with time (Supplementary Figure S8D).

Collagen VI expression was barely observed in the quadriceps at 20 weeks following transplantations at the neonatal stage and boost transplantation at 4 weeks (Supplementary Figure S10A). The longest period the donor cells could engraft was not confirmed, but some donor cells were detected in the quadriceps at 20 weeks (Supplementary Figure S9B). No tumorigenesis was confirmed at 20 weeks in any of the 10 mice that received the two transplantations (neonatal and 4-week boost) (data not shown).

### iMSC Transplantation Activated Muscle Regeneration

We then focused on the impact of iMSC transplantation on MYH3+ myofibers. MYH3, an embryonic myosin heavy chain, is the isoform expressed in regenerating fibers after injury or the early stage of muscle development (Schiaffino et al., 2015) and generally detected for 2–3 weeks in newly regenerating myofibers (Jerkovic et al., 1997; Kalhovde et al., 2005). The MYH3 protein expression level gradually decreases and switches to adult-type myosin heavy chain (Li et al., 2013; Yoshimoto et al., 2020). The position change of nuclei is another hallmark of muscle development (Roman and Gomes, 2018). During development, nuclei are spread longitudinally in the center of the myofiber and migrate peripherally toward the end part of the muscle (Roman and Gomes, 2018), which can be observed as a multinuclear myofiber in the section. In a previous study using muscle biopsies from UCMD patients, MYH3 positive regenerating muscle fibers were limited to small diameters (Higuchi et al., 2003), indicating that the impairment of muscle regeneration is one of the phenotypes of UCMD.

**FIGURE 3:**
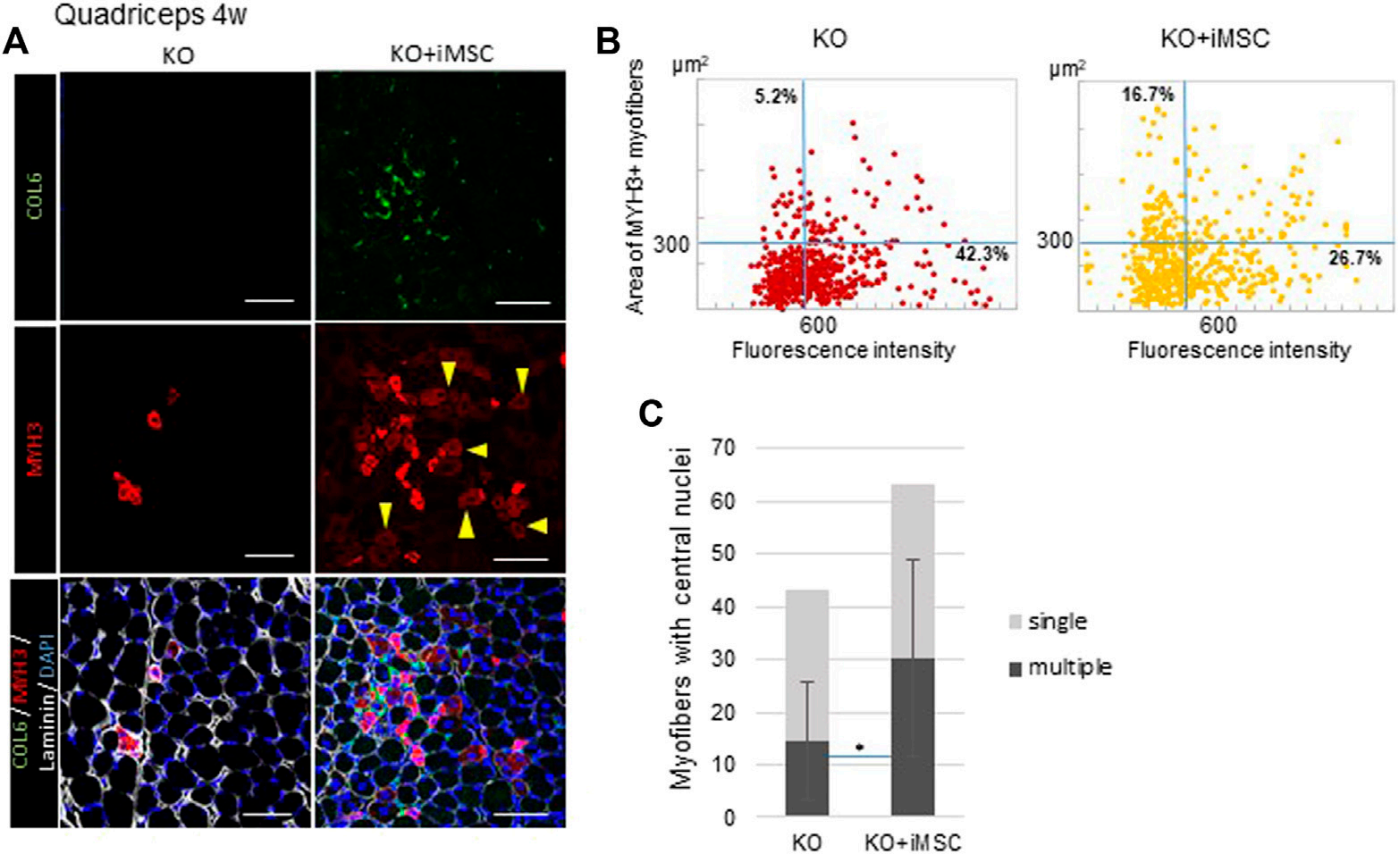
Regenerating myofibers and collagen VI at 4 weeks. **(A)** Immunofluorescence staining of the quadriceps from non-transplanted mice (left) and transplanted mice (right). MYH3+ myofibers were gathered and enlarged around the area where collagen VI was expressed in the transplanted mice. Some MYH3+ myofibers were faint in color (arrowheads), consistent with muscle maturation. Scale bars, 50 μm.**(B)** Correlation plot of the fluorescence intensity and area of MYH3+ myofibers in non-transplanted (left) and transplanted (right) mice. MYH3+ myofibers in the quadriceps of four mice were counted. The percentage in the upper left quadrants, which correspond to more mature MYH3+ myofibers, was increased with iMSC transplantation. **(C)** Number of central nucleated myofibers in the quadriceps. Myofibers with single nuclei (light grey) were not significantly different, but more myofibers with multiple nuclei (dark gray) were observed in the quadriceps in transplanted mice. KO (*n* = 9) and KO + iMSC (*n* = 9). (Student’s *t*-test). Error bars indicate ±SD.

At 4 weeks, we observed the area where MYH3+ myofibers were aggregated, which was consistent with the area where collagen VI was expressed in transplanted mice, while MYH3+ myofibers in non-transplanted mice solely existed among mature myofibers (Figure 3A). In addition, myofibers weakly positive for MYH3 observed in the transplanted mice grew (Figure 3A, arrowheads). Therefore, we analyzed the correlation of the fluorescence intensity and size of MYH3+ myofibers to evaluate the difference in muscle regeneration between transplanted and non-transplanted mice. The number of large, weakly positive MYH3+ myofibers was increased in transplanted mice, whereas smaller, strongly positive MYH3+ myofibers were observed in non-transplanted mice (Figure 3B). The number of myofibers with multiple nuclei as well as the ratio of multinucleated to single nucleated myofibers were increased in transplanted mice (Figure 3C).

**FIGURE 4:**
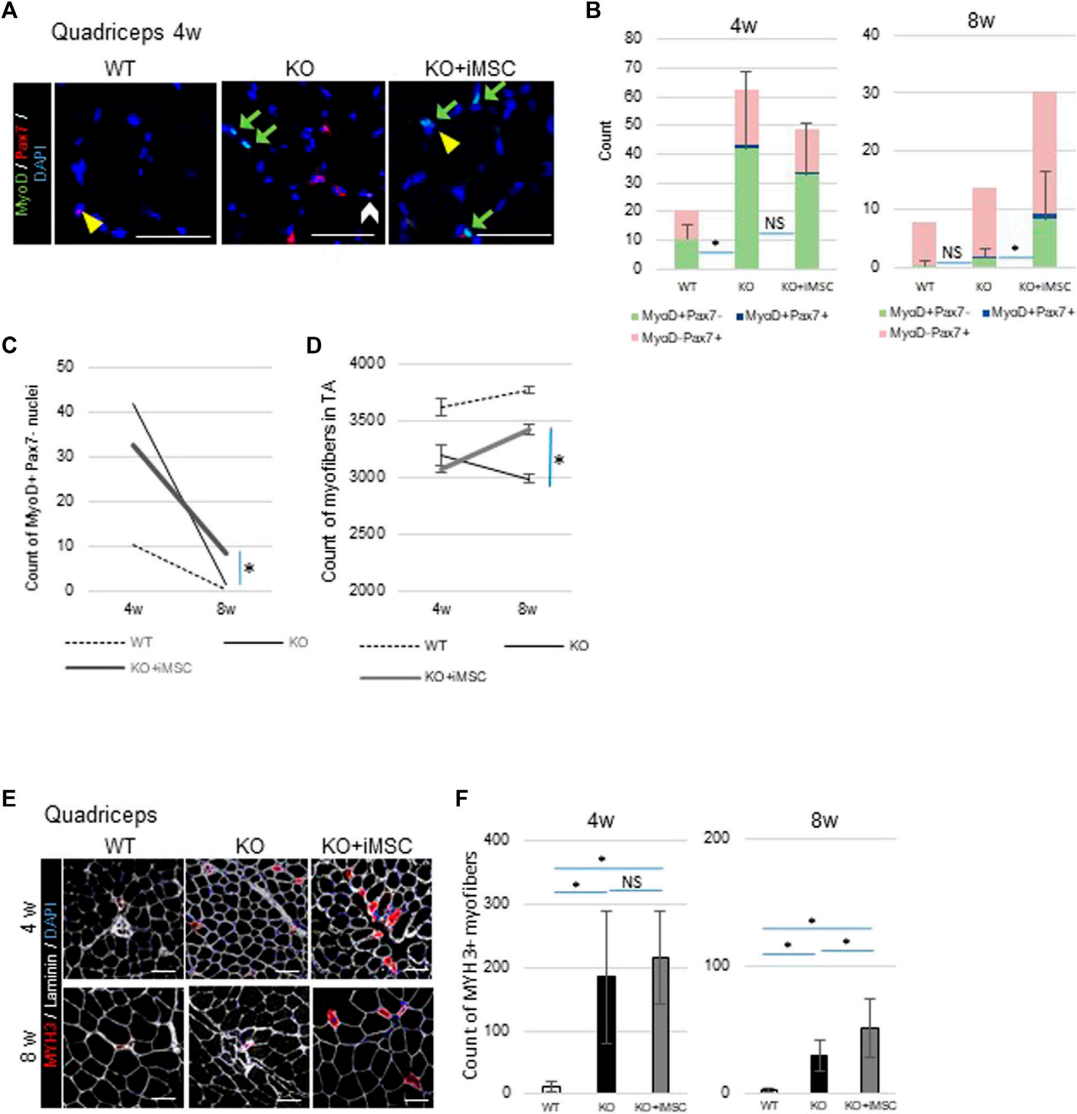
Histological analysis of muscle regeneration. **(A)** Representative images of immunostaining of nuclei with positive regenerative markers. MyoD + cells (green arrows), Pax7+ cells (yellow arrowheads), and MyoD + Pax7+ cells (white arrow) were observed in the quadriceps. Scale bars, 50 μm. **(B)** Quantitative analysis of muscle regeneration capacity at 4 weeks (WT (*n* = 4), KO (*n* = 11), and KO + iMSC (*n* = 11)) and 8 weeks (WT (*n* = 5), KO (*n* = 9), and KO + iMSC (*n* = 13)). The number of nuclei positive for each regenerative marker was counted (Tukey’s test). Error bars indicate ±SD. **(C)** Number of Myo + Pax7-nuclei between 4 and 8 weeks. A therapeutic difference was observed in transplanted mice at 8 weeks based on the number of MyoD + nuclei. **(D)** The number of myofibers in TA muscles between 4 and 8 weeks. 4 weeks: WT (*n* = 5), KO (*n* = 7), and KO + iMSC (n = 11); 8 weeks: WT (*n* = 5), KO (*n* = 8), and KO + iMSC (*n* = 13). (Student’s *t*-test). Error bars indicate ±SEM. **(E)** Images of MYH3+ myofibers at 4 and 8 weeks. **(F)** The number of MYH3+ myofibers in the quadriceps at 4 weeks (WT (*n* = 5), KO (*n* = 7), and KO + iMSC (*n* = 11)) and at 8 weeks (WT (*n* = 5), KO (*n* = 9), and KO + iMSC (*n* = 12)). (Tukey’s test). Error bars indicate ±SD. These results suggested iMSC transplantation positively affected regenerative capacity at 8 weeks.

Next, in order to reveal the impact of iMSC transplantation on muscle stem (satellite) cells, we counted the number of Pax7+ and MyoD + nuclei in the quadriceps (Figure 4A). Pax7 is a marker of satellite cells, and Pax7+MyoD-cells are in the quiescent state (Gnocchi et al., 2009). On the other hand, MyoD is one of the earliest markers of myoblasts (Weintraub, 1993). Pax7+MyoD + cells are active satellite cells during the process of cell division, and MyoD + Pax7-cells are committed myoblasts (Chal and Pourquié, 2017). Muscle regeneration was more active at 4 weeks than at 8 weeks, during which time the total number of positive nuclei for these regeneration markers decreased in both WT/NSG and *Col6a1* ^*KO*^/NSG mice (Figure 4B). A difference in the number of MyoD + Pax7-cells was notable at 8 weeks between non-transplanted and transplanted mice. MyoD + Pax7-cells were upregulated in the non-transplanted mice up to 4 weeks, but thereafter decreased to a level close to WT/NSG mice at 8 weeks. Contrastingly, the count of MyoD + Pax7-cells in the transplanted mice decreased at a slower rate at 8 weeks than in the non-transplanted mice (Figures 4B,C). The total number of myofibers in TA muscle increased in transplanted mice as well as in WT/NSG mice, while it was decreased in non-transplanted mice from 4 to 8 weeks (Figure 4D).

The dynamics of MYH3+ regenerating myofibers followed the dynamics of MyoD + cells. While MYH3+ myofibers were rarely observed in WT/NSG mice at 4 and 8 weeks, they were scattered in both transplanted and non-transplanted *Col6a1* ^*KO*^/NSG mice at 4 weeks. However, at 8 weeks, larger MYH3+ myofibers were present in the transplanted mice but rarely detected in the non-transplanted mice (Figure 4E). The number of MYH3+ myofibers was not different between the transplanted and non-transplanted mice at 4 weeks, but higher in the transplanted mice at 8 weeks (Figure 4F).

In sum, the dynamics of myogenic cells and MYH3+ myofibers indicated that iMSC transplantation retained muscle regenerative capacity over longer periods in *Col6a1* ^*KO*^/NSG mice.

### Effects on Mitochondria Morphology and Apoptosis

Ultrastructural alterations of mitochondria and spontaneous apoptosis are observed in the muscles of *Col6a1* ^*−/−*^ mice (Irwin et al., 2003; Palma et al., 2009) and UCMD patients (Angelin et al., 2007). Mitochondria are involved in the pathogenesis of UCMD, as mitochondrial defects are common findings irrespective of the genetic mutation locus or mode of inheritance, but additional factors are involved in the susceptibility to apoptosis or regeneration (Angelin et al., 2007). Therefore, to elucidate the mechanism of the therapeutic effects of iMSC transplantation, we investigated mitochondria and apoptosis in the muscles of *Col6a1* ^*KO*^/NSG mice.

**FIGURE 5:**
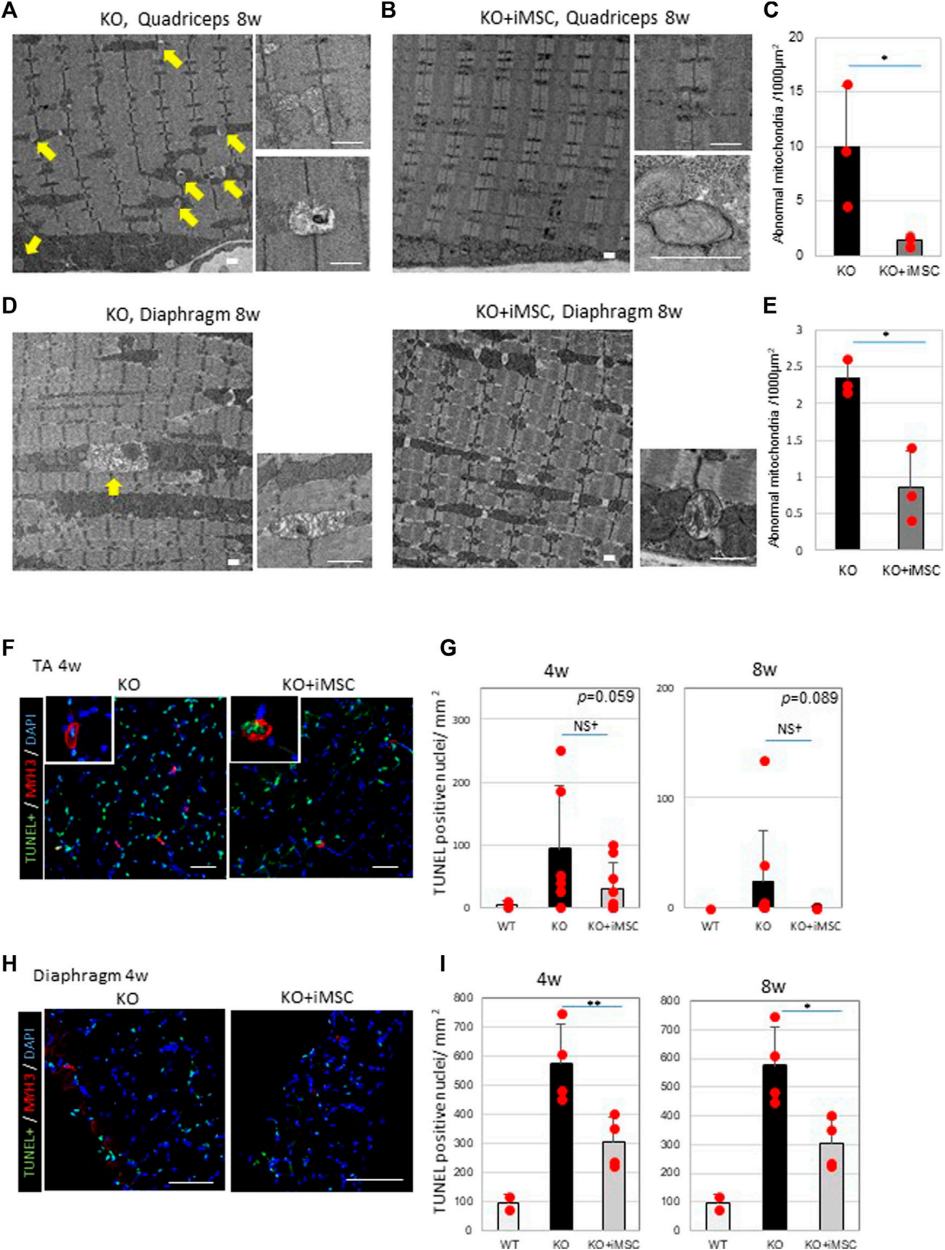
Abnormal mitochondria and apoptosis were decreased in the muscles of iMSC transplanted mice. **(A)** Electron micrographs of the quadriceps of *Col6* ^*KO*^/NSG mice without transplantation at 8 weeks. Abnormal mitochondria (arrows) are indicated (left). A typical picture of aberrant mitochondria revealed swollen bodies (top right), some of which were rich with protein aggregates (bottom right). Scale bars, 1 μm. **(B)** Electron micrographs of the quadriceps of transplanted KO mice at 8 weeks. Mitochondrial abnormalities were rarely detected (left). Normal mitochondria (top right) and the formation of the autophagosome (bottom right) were also observed. Scale bars, 1 μm. **(C)** Quantification of abnormal mitochondria in the quadriceps at 8 weeks. (Student’s *t*-test). **(D)** Electron micrographs of the diaphragms from non-transplanted (left) and transplanted (right) mice at 8 weeks. Abnormal mitochondria were present in non-transplanted mice (arrow), but less so in transplanted mice. Swollen mitochondria were detected in both non-transplanted and transplanted mice (bottom right). Scale bars, 1 μm. **(E)** Quantification of abnormal mitochondria in the diaphragm at 8 weeks. (Student’s *t*-test). **(F)** Immunofluorescence staining of the TUNEL assay and MYH3 from TA muscles at 4 weeks. Pictures at the top left are of MYH3+ myofibers whose nuclei were apoptotic, indicating that newly regenerating myofibers were undergoing apoptosis. Scale bars, 50 μm.**(G)** Quantification of apoptotic nuclei in TA muscles at 4 weeks (WT (n = 2), KO (n = 7) and KO + iMSC (*n* = 9)) and 8 weeks (WT (*n* = 2), KO (*n* = 8) and KO + iMSC (*n* = 8)). (Student’s *t*-test). **(H)** Immunofluorescence staining of the TUNEL assay and MYH3 from the diaphragm at 4 weeks. Scale bars, 50 μm. **(I)** Quantification of apoptotic nuclei in the diaphragm at 4 weeks (WT (*n* = 2), KO (*n* = 4), and KO + iMSC (*n* = 4)) and 8 weeks (WT (*n* = 2), KO (*n* = 5), and KO + iMSC (*n* = 4)). (Student’s *t*-test). All error bars indicate ±SD.

Regarding the morphology of the mitochondria, multiple abnormal mitochondria were often detected in the quadriceps of *Col6a1* ^*KO*^/NSG mice (Figure 5A), but they were hardly observed in transplanted mice (Figure 5B). Moreover, some images of the transplanted mice were consistent with the formation of the autophagosome (Figure 5B), which was not the case in non-transplanted mice. The number of abnormal mitochondria was reduced in the quadriceps (Figure 5C). Abnormal mitochondria were observed in the diaphragm of both non-transplanted and transplanted mice (Figure 5D), but they were fewer in the transplanted group (Figure 5E).

Apoptotic nuclei were counted in the TA muscles and diaphragm (Figures 5F,H). The number of apoptotic cells tended to be reduced in the TA muscles of transplanted mice (Figure 5G), and less apoptosis was identified in the diaphragm of the same group (Figure 5I). MYH3+ myofibers were simultaneously stained to evaluate the correlation between apoptosis and regenerating myofibers. Some TUNEL positive nuclei were observed in the MYH3+ myofibers in the TA muscles of both non-transplanted and transplanted mice at 4 weeks (Figure 5F), possibly reflecting the specific pathology of UCMD muscles, in which new myofibers during regeneration fail to grow and mature, undergoing apoptosis instead (Paco et al., 2012). On the contrary, TUNEL + MYH3+ myofibers were not detected in the transplanted mice at 8 weeks (data not shown) or at 4 weeks in the diaphragm (Figure 5H). Single-stranded DNA-positive nuclei were examined as an alternative marker for apoptosis. A similar distribution of single-stranded DNA positive nuclei was confirmed in the quadriceps and diaphragm of both non-transplanted and transplanted mice (Supplementary Figure S10).

These results confirmed abnormal mitochondria and apoptosis in *Col6a1* ^KO^/NSG mice at 4 and 8 weeks and that iMSC transplantation reduced the number of dysmorphic mitochondria and apoptotic cells.

### iMSC-Transplanted Mice Were Functionally Improved at 8 weeks

**FIGURE 6:**
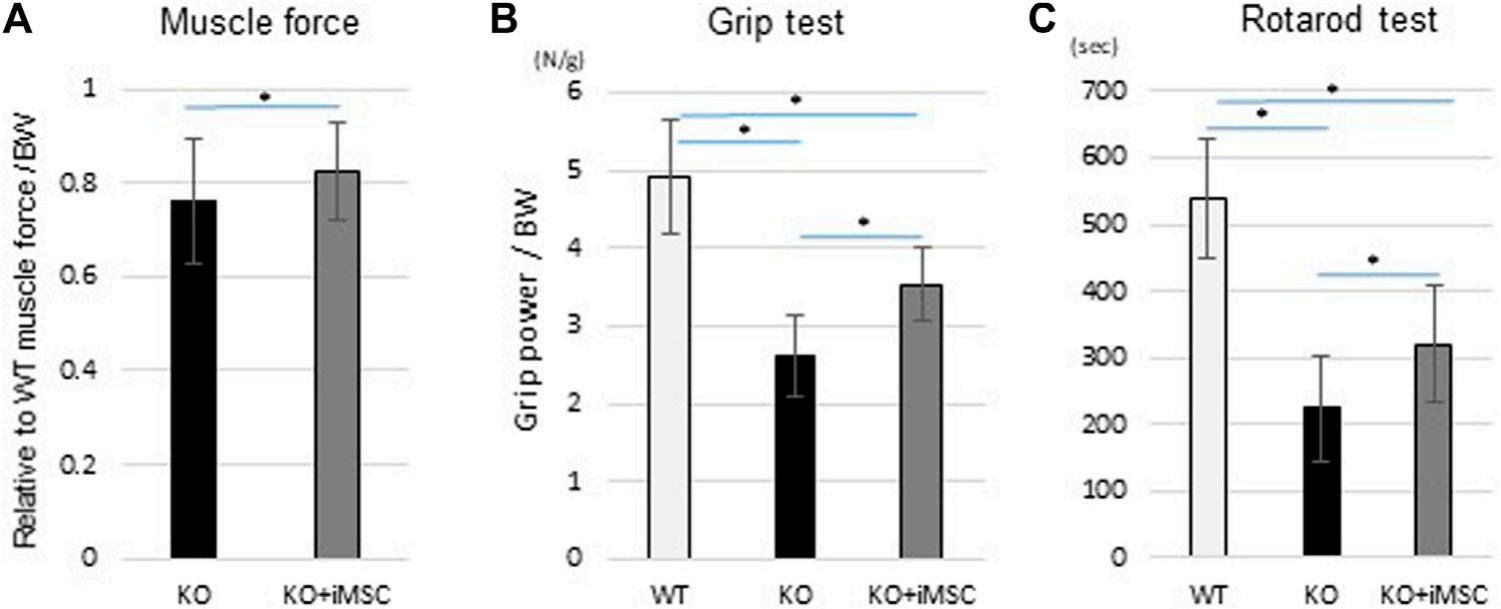
Motor functional tests at 8 weeks. The same mice conducted three different functional tests. KO (*n* = 13) and KO + iMSC (*n* = 14). **(A)** Maximum isometric contraction was measured from both sides of the gastrocnemius under anesthesia. Values are relative to WT mice (*n* = 4) and normalized by body weight. (Student’s *t*-test). **(B)** Grip strength with the four limbs was measured three times in each mouse. The average value was used to evaluate improvement in the transplanted mice. (Tukey’s test). **(C)** The rotarod test showed tolerance at higher speeds in transplanted mice compared to non-transplanted mice. (Tukey’s test). All error bars indicate ±SD.

According to a previous report, 14-week-old *Col6a1* ^*KO*^ mice showed functional impairment in grip power, voluntary running, and muscle force (Noguchi et al., 2017). We compared the difference in motor functions in 8-week-old mice to evaluate if the transplantation therapy was functionally beneficial. Care was taken to minimize any environmental factors that might affect the results of the function tests (see Methods). Muscle force was measured with isometric contraction by both sides of the gastrocnemius muscle and was increased with iMSC transplantation (Figure 6A). Grip power was improved by 35% with the iMSC transplantation (Figure 6B). The average running time in the rotarod test was increased in the transplanted mice compared to the non-transplanted mice (Figure 6C). The percentage of mice that completed a 5-min run was 2.6% (1/30) in the non-transplanted group and 9.5% (4/42) in the transplanted group.

## Discussion

This study examined the therapeutic effects of systemic iMSC transplantation in UCMD model mice. We demonstrated that iMSCs act as a vector of collagen VI to skeletal muscles. The rescued collagen VI contributed to the upregulation of muscle regeneration and amelioration of the characteristic pathology of muscles in *Col6a1* ^*KO*^/NSG mice. Adding the better performance in motor function tests, our findings are preliminary evidence that systemic iMSC transplantation could be a feasible treatment option, especially for neonates or infants with Col6-deficient myopathy.

The muscle regeneration in *Col6a1* ^*KO*^/NSG mice was enhanced at 4 weeks but decreased at 8 weeks, and iMSC transplantation sustained muscle regeneration. Because our intervention was from the neonatal period, careful interpretation of the results is necessary when considering the effect of the collagen VI replacement, including its effects on muscle growth. Postnatal muscle growth results primarily from individual fiber hypertrophy, and the synthesis of new myotubes from myogenic stem cells contribute only a small part (Gokhin et al., 2008). The fusion of myogenic cells regenerates myofibers once the satellite cell niche is established during the neonatal period (Chal and Pourquié, 2017). Our results suggested that the supplementation of collagen VI promoted mainly muscle hypertrophy up to 4 weeks based on histological analysis, in which the size of the myofibers was increased by transplantation, but the number of myofibers was not. After 4 weeks, the primary effect of collagen VI was to sustain muscle regeneration. A previous study reported that collagen VI is a key component of the satellite niche and that it takes more time to regenerate injured skeletal muscle in *Col6a1* ^*−/−*^ mice than WT mice, though the experiments were performed with older mice (6 months old) (Urciuolo et al., 2013). Our experiments indicated that the collagen VI effects depends on the stage of muscle growth and development. Neonatal transplantation can mimic the physiological condition by supplying collagen VI from the early period of life, which our findings suggest has a positive impact on muscle development. Eventually, the hypertrophy during muscle growth and maintained regeneration led to muscle weight gain and better motor function at 8 weeks. These observations suggest that there is a critical time point for collagen VI supplementation to induce its maximum effects on muscle regeneration.

Notably, we found that the restoration of collagen VI rescued the removal of aberrant mitochondria and possibly suppressed apoptotic events in the transplanted mice. Furthermore, apoptotic cells were rarely observed in the muscle of transplanted mice at 8 weeks. These results are consistent with several previous reports that showed defective mitochondria and ensuing apoptosis in *Col6a1*-null muscles and that pharmacological interventions such as cyclosporine A or other cyclophilin inhibitors, which inhibit the mitochondrial permeability transition pore (PTP), normalized both pathologies, although the mechanism is still not understood (Irwin et al., 2003; Angelin et al., 2007; Merlini et al., 2008; Hicks et al., 2009).

We estimated that the amount of collagen VI rescued by iMSC transplantation was less than 3% total collagen VI in WT/NSG mice. Despite this low level, some pathologies were improved, including mitochondrial morphology, the suppression of cellular apoptosis, and muscle regeneration. We, therefore, speculated that collagen VI might act as a signal transmitter in skeletal muscles in addition to acting as an extracellular matrix. This assumption could explain why low levels of collagen VI could ameliorate the cellular phenotypes. Recently, collagen V has been elucidated as a signal transmitter via the calcitonin receptor in muscle satellite cells (Baghdadi et al., 2018). Although previous studies have shown that integrin β1 and the membrane-spanning proteoglycan NG2 (neuron/glia antigen 2) are receptors for collagen VI (Doane et al., 1998; Tillet et al., 2002; Cattaruzza et al., 2013), it is still unknown whether these molecules are involved in the homeostasis of satellite cells or myofibers.

Notably, the systemic injection used in this study did not cause invasive damage to the muscles. Two previous studies (Alexeev et al., 2014; Takenaka-Ninagawa et al., 2021) that injected adipose-derived MSCs or iMSCs into TA muscles found the muscle may be damaged by the injection, which in turn may affect the regeneration. Furthermore, those studies injected the cells in adult mice, while our injections were done in neonatal mice. The timing of the injections was also different.

Additionally, we performed two transplantations, one at the neonatal stage and the other at 4 weeks, since neither transplantation alone was sufficient for therapeutic effects. Mice who had only the neonatal transplantation did not show a gain in muscle weight at 8 weeks, nor did they show functional improvements. When only one transplantation was performed at 4 weeks, the impact on muscle regeneration was not significant, and there were no changes in the number of MYH3+ myofibers in the quadriceps (data not shown). These results indicate the effectiveness of the boost transplantation and importance of neonatal intervention for the therapeutic effects in our model.

iMSCs are not the only candidate for systemic cell therapy to treat UCMD or other COL6-related myopathies; primary MSCs from other tissues are theoretically applicable, since they too secrete collagen VI. The definitive advantage of using iMSCs, however, is the large number of cells with homogeneous quality that can be prepared for the transplantation. iMSCs have a stable proliferative capacity up to at least 6 passages (Chijimatsu et al., 20172017). Although the risk of oncogenesis remains when using iPSC-derived cells, tumorigenesis was not observed up to 20 weeks in our experiments or 24 weeks when iMSCs were injected intramuscularly (Takenaka-Ninagawa et al., 2021). Our results also indicated that a small number of iMSCs persistently remained in both muscles and non-muscle tissues for longer periods, suggesting longer observation times are required to assess safety. Furthermore, ongoing research around iPSC technology may allow us to apply immunologically favorable cells that are less susceptible to HLA sensitization, such as HLA-homozygous iPSC line stocks (Gourraud et al., 2012; Karagiannis et al., 2019) or the generation of HLA-C-retained/HLA-class II knockout iPSCs (Xu et al., 2019). Considering that repetitive transplantations will be necessary in the clinical setting, it is crucial to have a cell source from the same donor with immunological properties identical to the patient.

The limitation of the study includes the i. p. transplantation, which is not a realistic route for cell transplantation in humans. I. v. was more efficient than i. p transplantation in terms of delivering the donor cells to the skeletal muscles; therefore, similar or better therapeutic effects can be expected with i. v. transplantation. The current study, however, is a proof of concept that iMSC transplantation can systemically produce small amounts of collagen VI to ameliorate the pathophysiology of *Col6a1* ^*KO*^/NSG mice. Further confirmation with larger animals will be necessary before clinical application in patients with COL6-related myopathies.

In a broader prospective, the present study demonstrated that iMSCs can supply deficient molecules to target organs, warranting the concept of stem cell therapies for some inherited disorders. The efficacy of stem cell therapies for supplying deficient molecules has been already reported in other congenital diseases such as osteogenesis imperfecta (Guillot et al., 2008; Götherström et al., 2014).

## Conclusion

We demonstrated the definite therapeutic effects of neonatal iMSC transplantation for immunodeficient UCMD model mice. Because iMSCs were able to migrate to the major organs, the supplementation of collagen VI was realized in skeletal muscles systemically. Accordingly, the histological and functional phenotypes specific to UCMD were ameliorated until at least 8 weeks after the transplantation in mice.

## Data Availability

The raw data supporting the conclusion of this article will be made available by the authors, without undue reservation.
