# Supplementary material for: Systemic Supplementation of Collagen VI by Neonatal Transplantation of iPSC-Derived MSCs Improves Histological Phenotype and Function of Col6-Deficient Model Mice
Source: Front Cell Dev Biol. 2021 Nov 23;9:790341. doi: 10.3389/fcell.2021.790341 (PMC8649773; doi:10.3389/fcell.2021.790341)
Supplement: Supplementary file 1 [file DataSheet1.pdf]

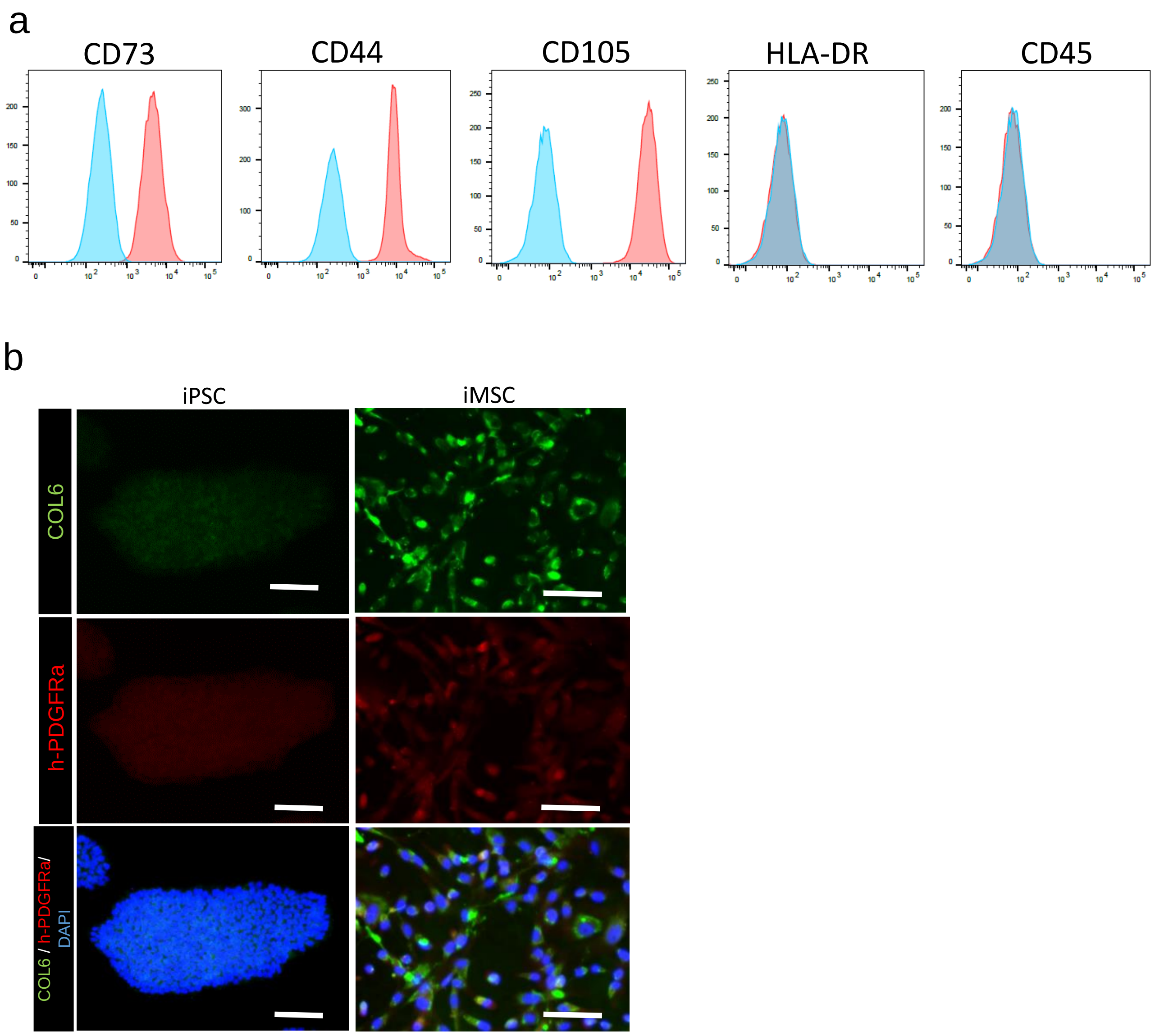

Supplementary figure 1. Characterization of donor iMSCs. a) Surface markers of iMSCs (passage 5). iMSCs were positive for CD 73, CD44 and CD105 and negative for HLA-DR and CD45. Negative control (blue) are isotype controls. **b)** Immunofluorocent staining. Donor cells expressed collagen VI, and human-PDGFRa was weakly expressed. Scale bars, 100 µm.

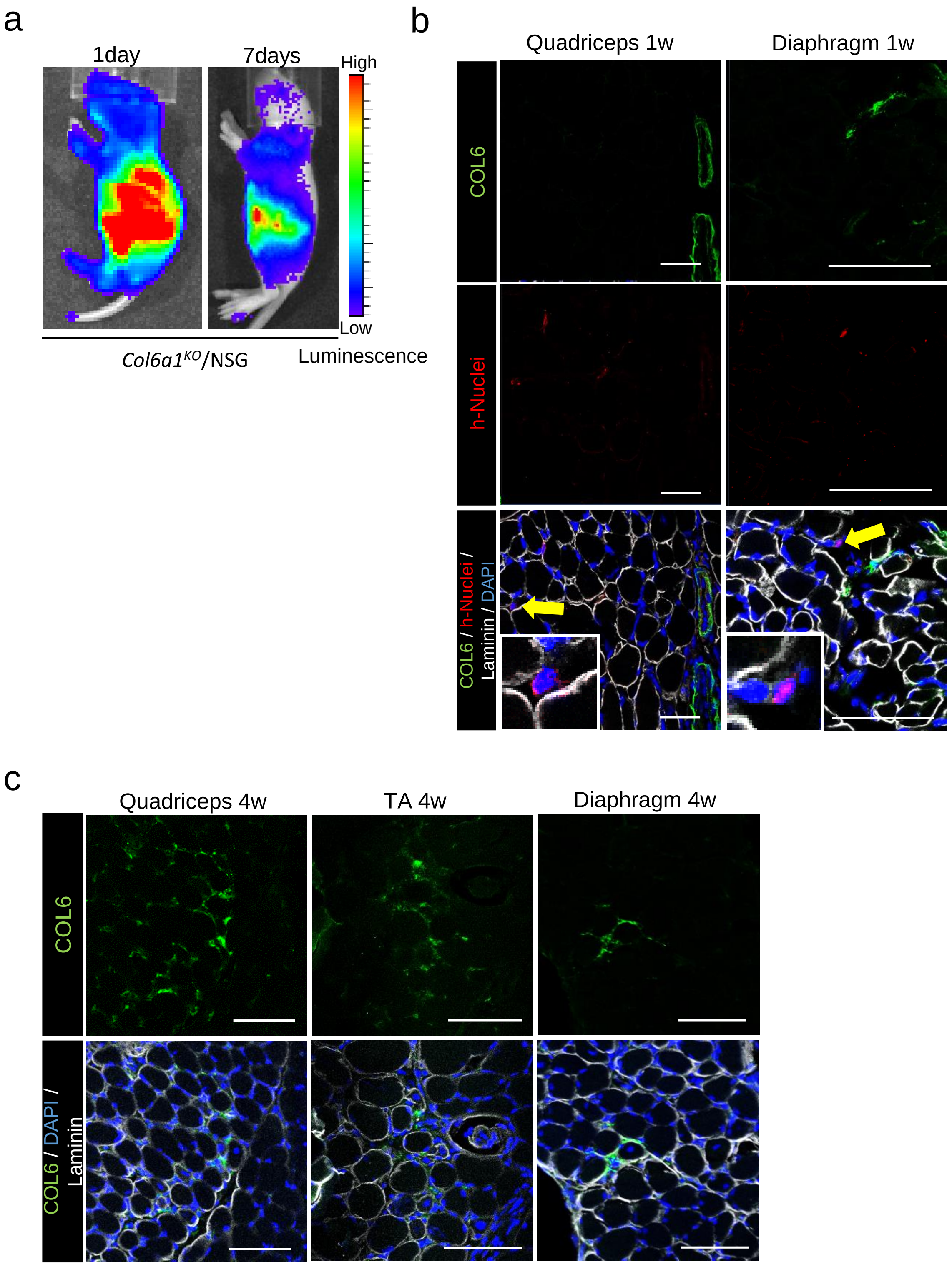

Supplementary figure 2. Dynamics of iMSCs after i.v. injection. a) Luciferase detection by IVIS. The images were taken 24 hours (left) and 7 days (right) after the transplantation. 5 x 10^5 iMSCs were intravenously injected from the facial vein. **b)** Immunohistochemistry of the quadriceps (left) and diaphragm (right) 1 week after the transplantation. Donor cells were detected in the quadriceps and diaphragm. Scale bars, 50 µm. **c)** Immunohistochemistry of the skeletal muscles at 4 weeks. Scale bars, 50 µm. These results suggest iMSCs distributed in the skeletal muscle *via* blood vessels.

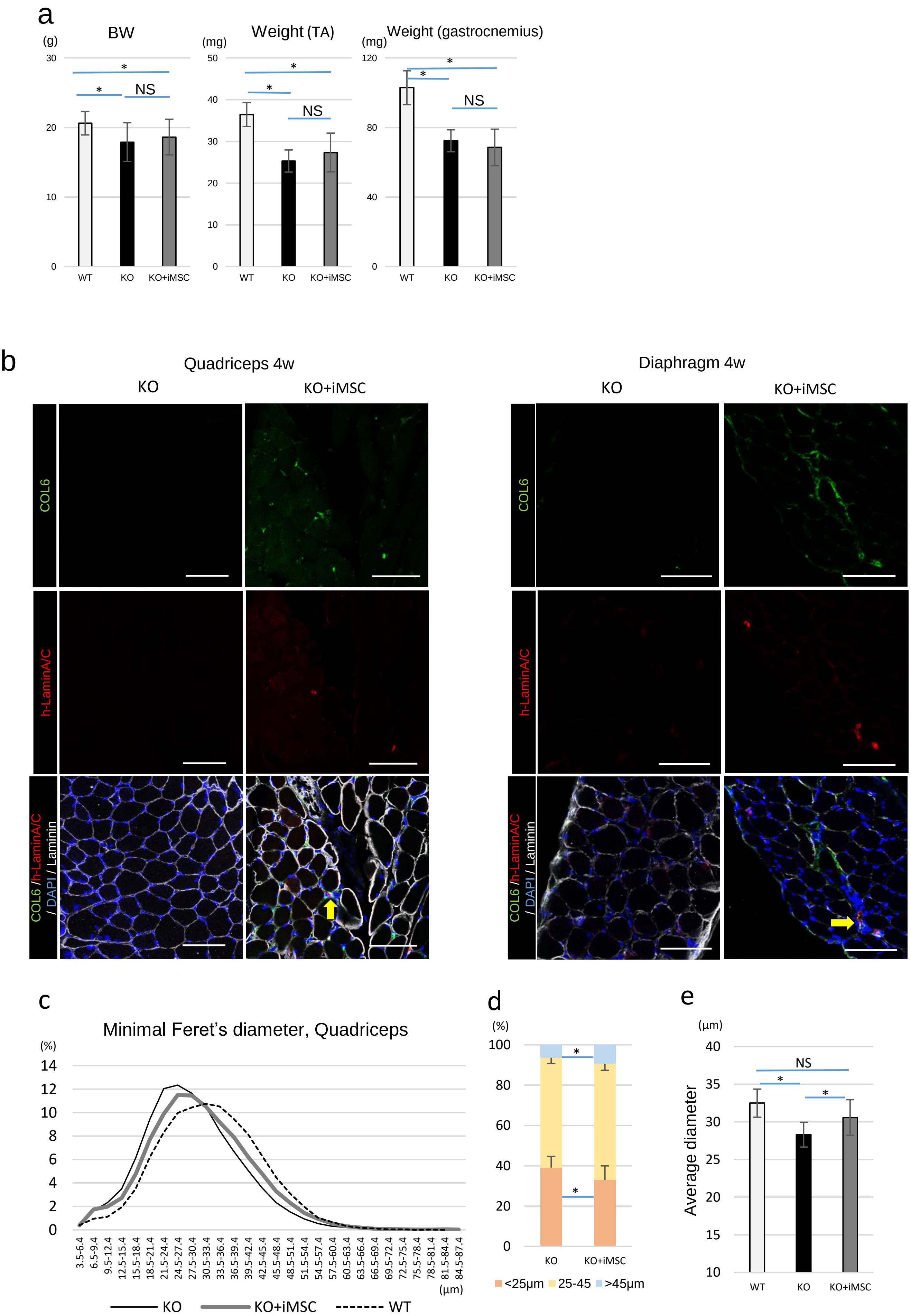

a
BW
(g)
30
20
10
0
*
*
NS
WT
KO
KO+iMSC
Weight (TA)
(mg)
50
40
30
20
10
0
*
*
NS
WT
KO
KO+iMSC
Weight (gastrocnemius)
(mg)
120
80
40
0
*
*
NS
WT
KO
KO+iMSC
b
Quadriceps 4w
KO
KO+iMSC
Diaphragm 4w
KO
KO+iMSC
COL6
h-LaminA/C
COL6 /h-LaminA/C / DAPI / Laminin
c
Minimal Feret's diameter, Quadriceps
(%)
14
12
10
8
6
4
2
0
3.5-6.4
6.5-9.4
9.5-12.4
12.5-15.4
15.5-18.4
18.5-21.4
21.5-24.4
24.5-27.4
27.5-30.4
30.5-33.4
33.5-36.4
36.5-39.4
39.5-42.4
42.5-45.4
45.5-48.4
48.5-51.4
51.5-54.4
54.5-57.4
57.5-60.4
60.5-63.4
63.5-66.4
66.5-69.4
69.5-72.4
72.5-75.4
75.5-78.4
78.5-81.4
81.5-84.4
84.5-87.4
(µm)
KO
KO+iMSC
WT
d
(%)
100
80
60
40
20
0
*
*
KO
KO+iMSC
<25µm
25-45
>45µm
e
(µm)
40
35
30
25
20
15
10
Average diameter
NS
*
*
WT
KO
KO+iMSC

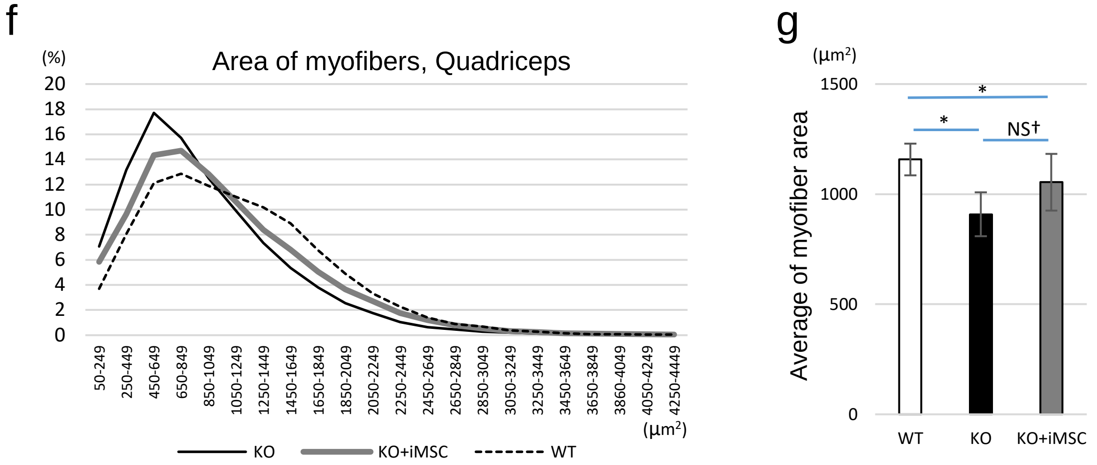

Supplementary figure 3. Phenotypes at 4 weeks. a) Body weight (BW) and muscle weight of each group. For BW: WT (n = 17), KO (n = 82) and KO+iMSC (n = 64). For raw weight of the TA (middle) and gastrocnemius muscles (right): WT (n = 6), KO (n = 11) and KO+iMSC (n = 15). (Tukey's test). **b)** Immunofluorescence staining of the quadriceps (left) and diaphragm (right) in a non-transplanted (KO) and transplanted mouse (KO+iMSC). Collagen VI expression remained at the basal lamina between myofibers. Engraftment of the donor cells is shown by the yellow arrows. Scale bars, 50 μm. **c)** Histogram of the short axial (minimal Feret's) diameter of myofibers in the quadriceps. WT (n = 6), KO (n = 10) and KO+iMSC (n = 12). **d)** Size pattern of the short axis in the quadriceps. (Student's *t*-test). **e)** Averaged short axial diameter in the quadriceps (Tukey's test). **f)** Histogram of the myofiber area in the quadriceps. **g)** Average myofiber area in the quadriceps. The myofiber area tended to increase in the transplanted mice compared with non-transplanted mice (p=0.064) (Tukey's test). All error bars indicate ± SD.

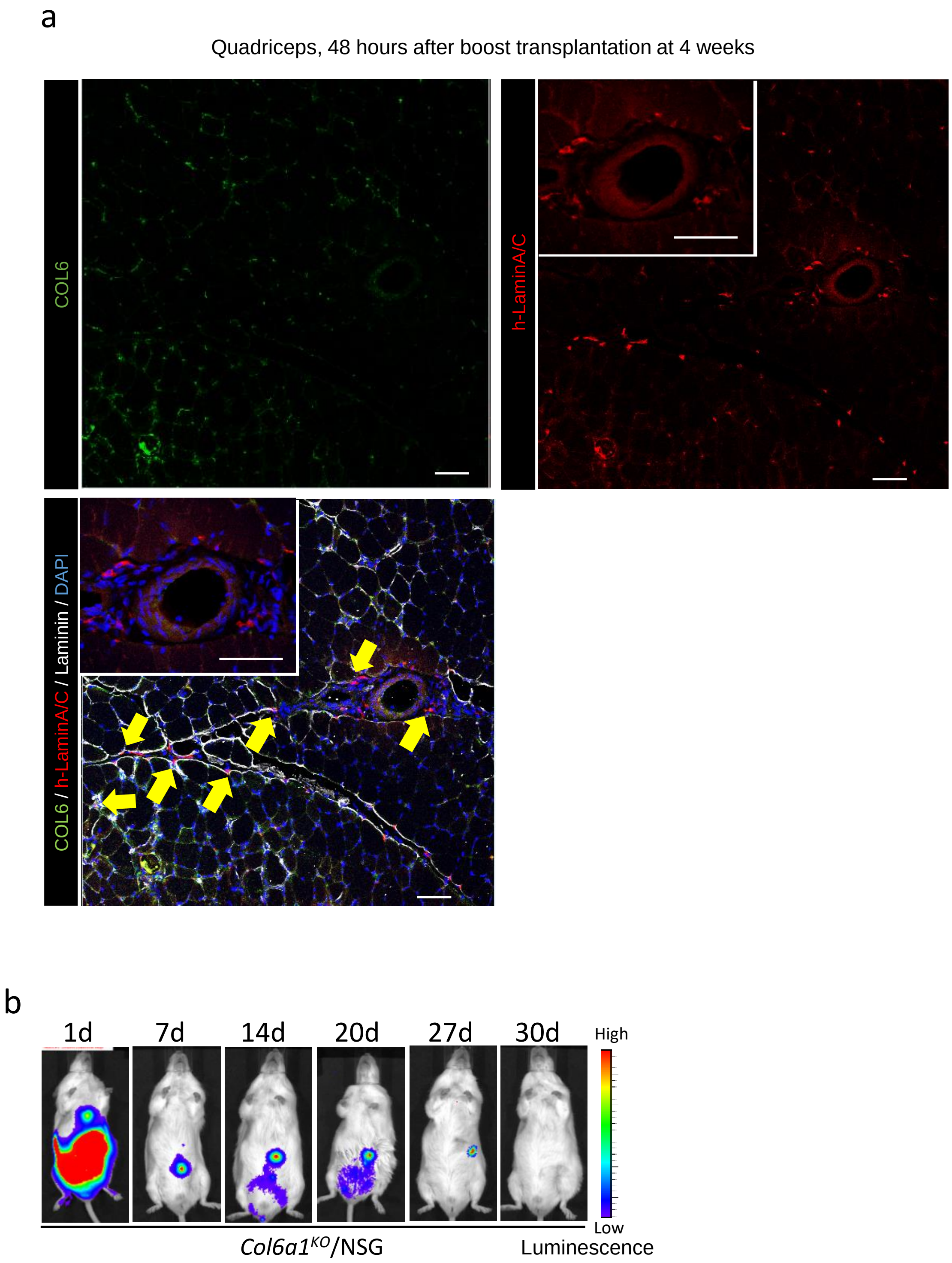

Supplementary figure 4. Tracking of donor cells after boost iMSC transplantation. a) Immunofluorescence staining of the quadriceps 48 hours after the boost transplantation at 4 weeks. Donor cells (yellow arrows) were present around the vessels and migrated along the perimysium. Scale bars, 50 µm. **b)** Luciferase detection of donor cells after the boost iMSC transplantation at 4 weeks.

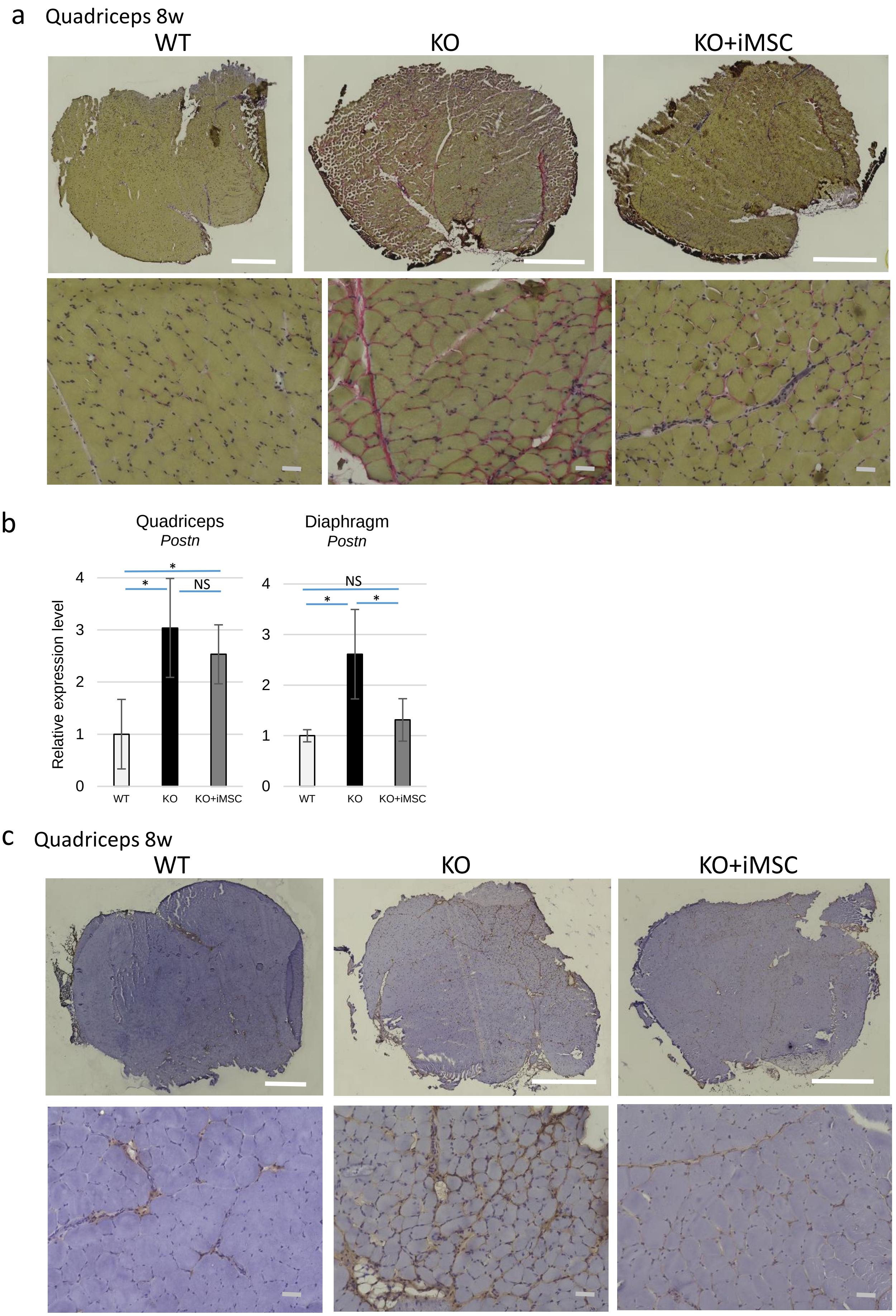

Supplementary figure 5. Assessment of fibrosis at 8 weeks. a) Sirius red staining of the quadriceps. **b)** The gene expression level of *Postn* in the quadriceps (left) and diaphragm (right). WT (n = 4), KO (n = 11) and KO+iMSC (n = 12). Error bars indicate ± SD. (Tukey's test). **c)** DAB staining for anti-Periostin antibody of the quadriceps. The bottom row is a magnification of the top row. Scale bars, 1000 μm for the top row and 50 μm for the bottom row.

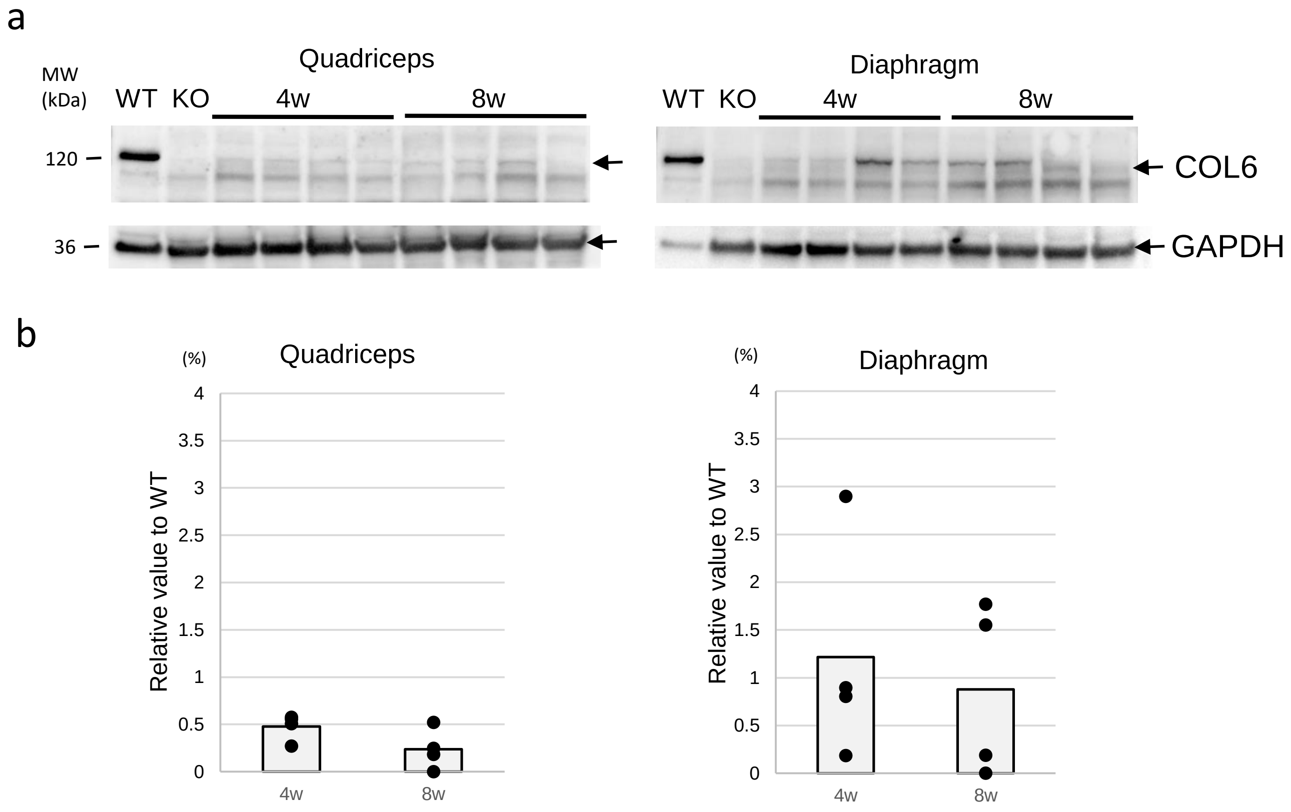

Supplementary figure 6. Quantification of collagen VI protein expression. a) Collagen VI and GAPDH in the quadriceps (left) and diaphragm (right) at 4 and 8weeks of the transplanted mice were quantified by western blotting. n=4 each. Positive controls were the quadriceps and diaphragm of WT mice at 4 weeks, respectively, with the aliquoted volume of the protein diluted by a factor of ten. All blots were derived from the same experiments and were processed in parallel. **b)** Relative values to WT (4 weeks) in the quadriceps (left) and diaphragm (right) were plotted. Grey bars indicate mean amounts of collagen VI at 4 and 8 weeks. Normalization of the values was performed using the concentration of protein lysates measured by the BCA assay.

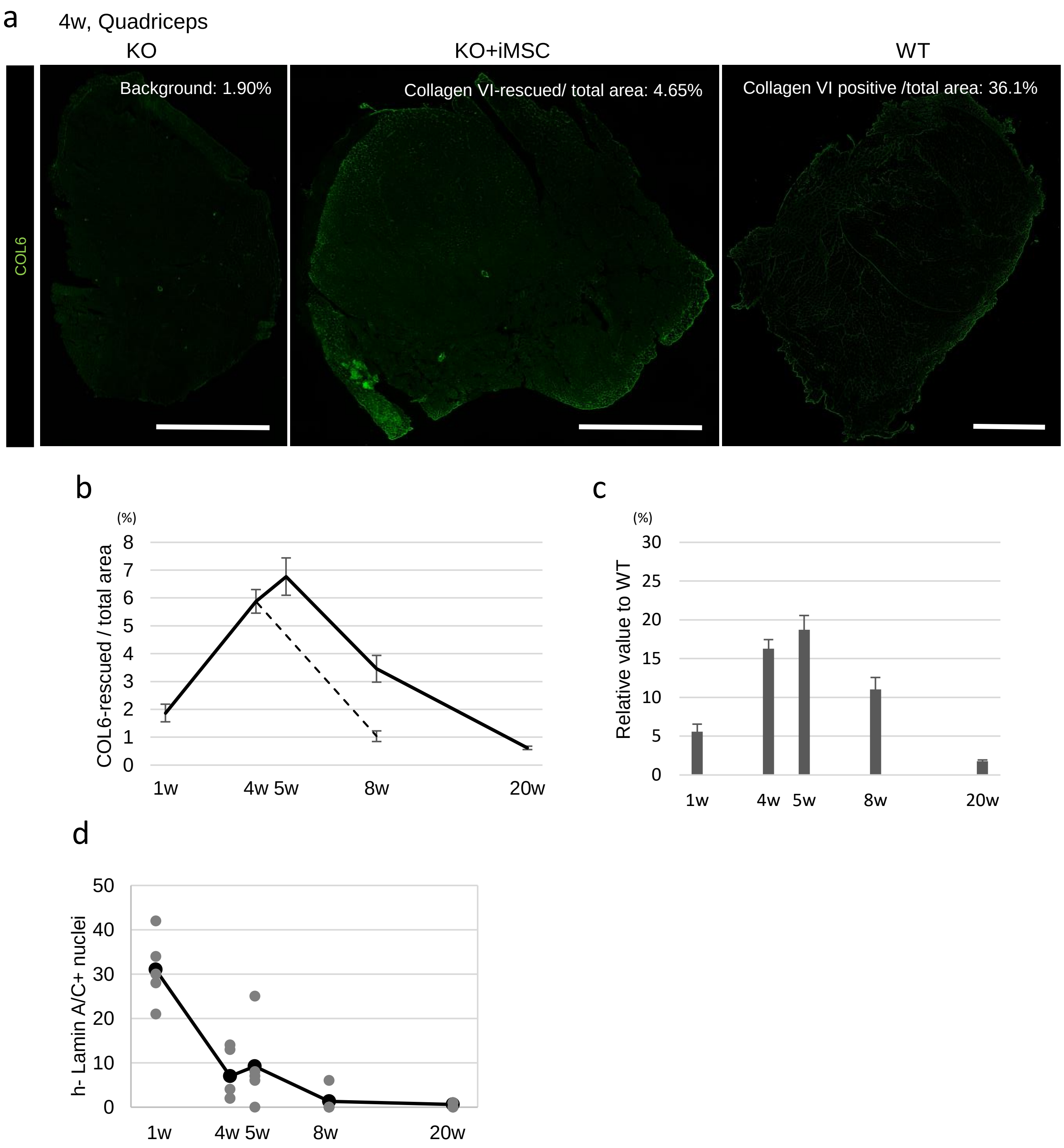

Supplementary figure 7. Quantification of the collagen VI-rescued area and donor cells in the quadriceps. a) Collagen VI immunostaining of the quadriceps at 4 weeks. Scale bars, 1000 μm. **b)** The ratio of the collagen VI-rescued area to total area in transplanted mice. The collagen VI-rescued area was calculated by subtracting the background values measured by the corresponding aged mice without iMSC transplantation. The solid line indicates the values when iMSCs were transplanted twice (neonatal and 4 weeks); the broken line indicates when iMSCs were transplanted once (neonatal). n = 5 in each group. **c)** The relative value of the collagen VI-rescued area in the transplanted mice to the collagen VI positive area in WT mice at each time point. **d)** Number of human-lamin A/C positive nuclei engrafted in the quadriceps. n=5 in each group. All error bars indicate ±SEM.

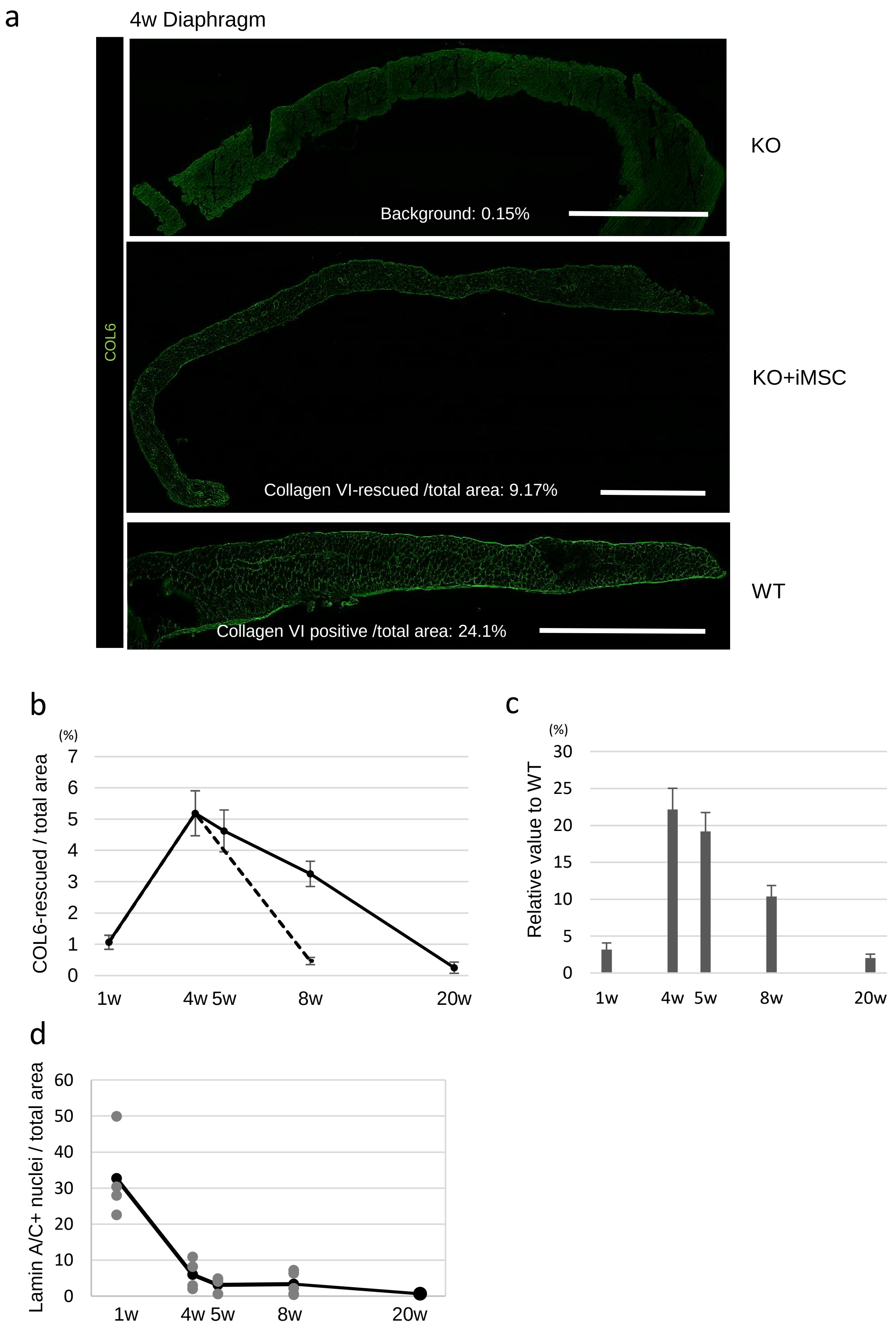

Supplementary figure 8. Quantification of the collagen VI-rescued area and donor cells in the diaphragm. a) Collagen VI immunostaining of the diaphragm at 4 weeks. Scale bars, 1000 μm. **b)** The ratio of the collagen VI-rescued area to total area in transplanted mice. The collagen VI-rescued area was calculated by subtracting the background values measured by the corresponding aged mice without iMSC transplantation. The solid line indicates the values when iMSCs were transplanted twice (neonatal and 4 weeks); the broken line indicates when iMSCs were transplanted once (neonatal). n = 4 in each group. **c)** The relative value of the collagen VI-rescued area in transplanted mice to the collagen VI positive area in WT mice. **d)** Number of human-lamin A/C positive nuclei engrafted in the diaphragm. n=4 in each group. All error bars indicate ±SEM.

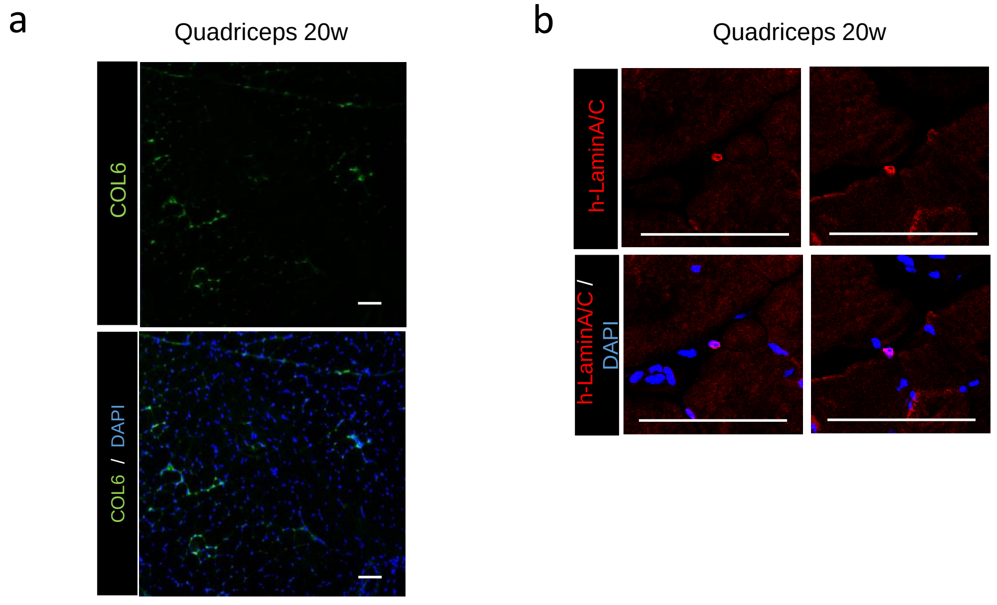

Supplementary figure 9. Long-term assessment of collagen VI expression and the engraftment of donor cells. a) COL6 staining of the quadriceps at 20 weeks after receiving two iMSC transplantations (neonatal and 4 weeks). Collagen VI was barely expressed. Scale bars, 50 µm. **b)** Human-lamin A/C staining of the quadriceps at 20 weeks after two iMSC transplantations (neonatal and 4 weeks). Scale bars, 50 µm.

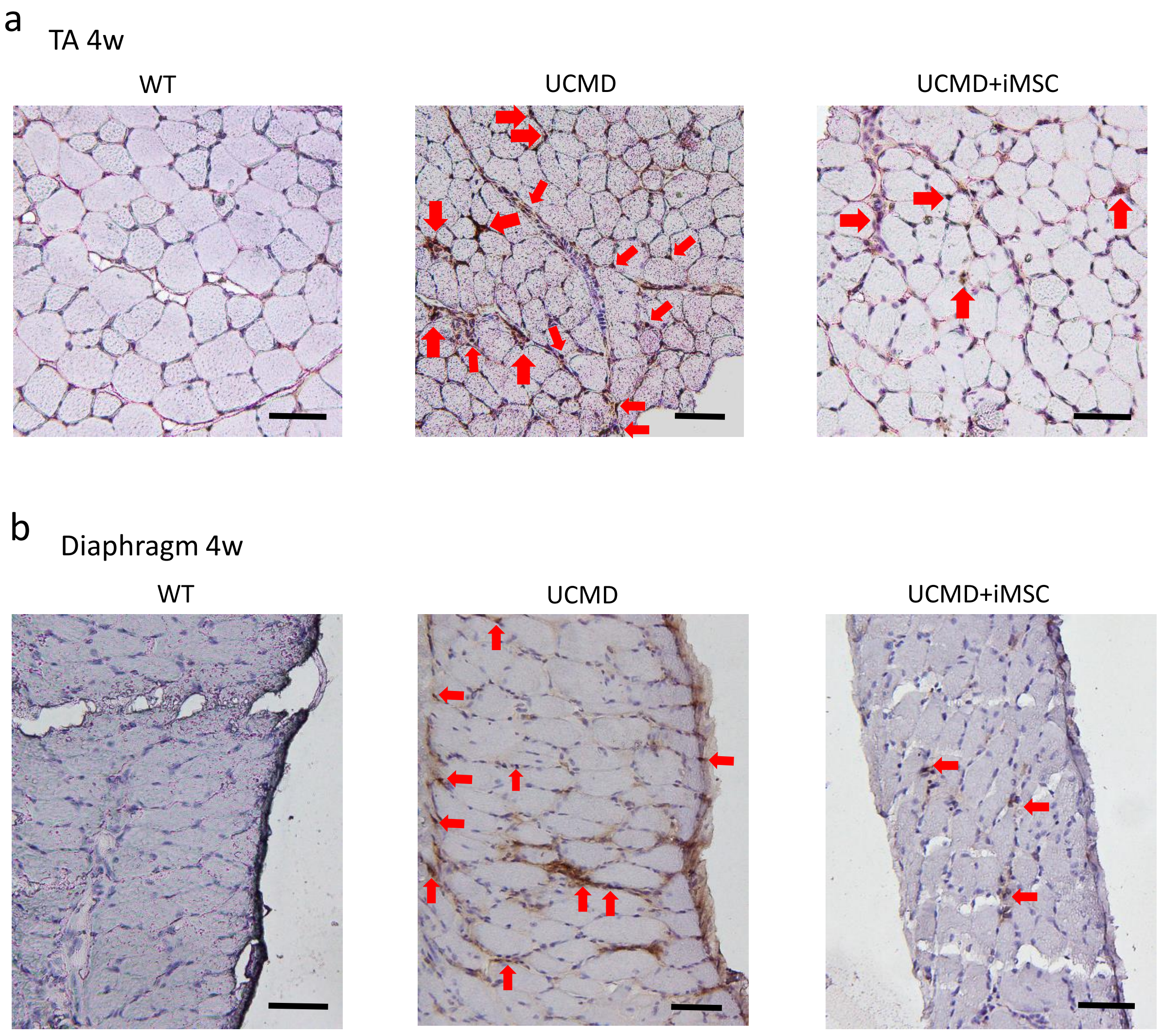

Supplementary figure 10. DAB staining for anti-single-stranded DNA antibody at 4 weeks. a) Single-stranded DNA, apoptosis marker, was detected in the quadriceps of both non-transplanted (middle) and transplanted (right) mice (red arrows). The shown samples for each muscle were stained simultaneously. **b)** Single-stranded DNA was positive in the diaphragm of non-transplanted (middle) and transplanted (right) mice (red arrows). The shown samples for each muscle were stained simultaneously. Scale bars, 50 μm.
