# Supplementary material for: Systemic Supplementation of Collagen VI by Neonatal Transplantation of iPSC-Derived MSCs Improves Histological Phenotype and Function of Col6-Deficient Model Mice
Source: Front Cell Dev Biol. 2021 Nov 23;9:790341. doi: 10.3389/fcell.2021.790341 (PMC8649773; doi:10.3389/fcell.2021.790341)
Supplement: Supplementary file 2 [file DataSheet2.DOC]

**Supplementary Materials and Methods**

**Neonatal intravenous transplantation**

For neonatal intravenous (i.v.) transplantation, approximately 5 x 10^5^ iMSCs were injected from the facial vein using a glass capillary tube (Narishige, GD-1, Tokyo, Japan) after processing using a puller (Narishige, PC-10, Tokyo, Japan).

**Sirius red staining**

Frozen samples on the slides were dehydrated with 100% ethanol (Nacalai tesque, CAS 08948-25) for 2 minutes and dried thoroughly, followed by fixation with 4% PFA for 2 minutes. The slides were dipped in 50% hematoxylin solution (Polyscience, 24901-250) for 3 minutes and washed with running water for 20 minutes. The Picrosirius Red Staining Kit (Polyscience, 24901-500) was used as instructed except that the incubation time of picrosirius red F3BA solution B was modified to 5 minutes.

**DAB staining**

Frozen sections of the samples were fixed with 1% PFA for 10 minutes at room temperature, washed with PBS twice, post-fixed in precooled ethanol and acetic acid (2:1 v/v) for 5 minutes at -20°C in a Coplin jar and washed again with PBS twice. Endogenous peroxidase was quenched with 3% hydrogen peroxide/PBS for 5 minutes at room temperature and rinsed in distilled water. The samples were incubated with primary antibodies, diluted in Can Get signal solution B, stored overnight at 4°C and washed with PBS for 5 minutes twice the next day. Anti-rabbit secondary antibody for Periostin (Histofine simple stain MAX-PO, NICHIREI, 414341) and anti-mouse secondary antibody for single-stranded DNA (Histofine simple stain MAX-PO, NICHIREI, 414322) were applied to the samples for 30 minutes at room temperature. The samples were then washed with PBS twice and incubated with DAB substrate (NICHIREI, 425011) for 5 minutes at room temperature. After washing, the samples were counterstained with hematoxylin (Merc, 1092490500) for 3 seconds and rinsed in water for 10 minutes. The primary antibodies used are summarized in Supplementary Table1.

**Histological analysis**

The number and size of the myofibers were counted with a microscope (Keyence, BZ-X700). Z-stack images were taken with a 20x objective lens and reconstructed on the X-Y axis to cover the whole piece of a sliced sample. Hybrid cell count software (Keyence, BZ-H3C) was used to recognize each myofiber as a bounded area. Area sizes between 50-8000 µm^2^ were included, and the area filling the interstitial space was removed manually. MYH3+ myofibers and TUNEL+ cells were imaged and analyzed with the same microscope and software by Keyence.

To calculate the restored collagen VI area, optical sectioning images were taken with a 10x objective lens and reconstructed on the X-Y axis. The background signal was suppressed with image processing for haze removal. The collagen VI positive and total areas in WT/NSG mice and non-transplanted and transplanted model mice were measured using BZ-H3C. The collagen VI restored area was calculated by subtracting the ratio of the Col6 positive area in non-transplanted mice from that in transplanted mice.

Because a higher resolution was necessary to count Pax7+ and MyoD+ cells, the images were taken with a confocal microscope (Zeiss, LSM710). A 3 x 3 tile scan covering a 225 µm x 225 µm area and two tile scan pictures per quadriceps were recorded with a frame size of 1024 x 1024 pixel and averaging number of 2. The tile scan pictures did not overlap each other and were randomly chosen. Pax7+ and MyoD+ single and double positive cells were counted manually.

**Flow cytometry**

5 x 10^5^ iMSCs (passage number 5) in 50 μL were incubated with antibodies for 30 minutes at 4°C in the dark and suspended with 1 mL FACS buffer. After re-suspending with 500 μL FACS buffer and filtration, the prepared cells were analyzed using a BD FACSAria^TM^ II cell sorter (BD Biosciences). The antibodies used and their dilutions were PE-CD44 (1:50) (BD Biosciences 550989), PE-CD73 (1:50) (BD Biosciences 550257), APC-CD105 (1:50) (eBioscience,17-1057-42), APC-H7 HLA-DR (1:20) (BD Biosciences 641393), APC-CD45 (1:50) (BD Biosciences 340943), Isotype APC (1:50) (BD Biosciences 565381) and Isotype PE (1:50) (BD Biosciences 551438).

**Immunocytochemistry**

Cells plated on 6-well microplates (Iwaki, 3810-006) were fixed with 2% paraformaldehyde for 15 minutes and washed with PBS twice. After the incubation with Blocking one for 1 hour, primary antibodies diluted with Can Get Signal solution B were applied to the cells, which were then incubated overnight at 4°C on a gentle shaker and washed with PBST 4 times the next day. Second antibodies were added for 1 hour at room temperature and washed 3 times with PBS. Images were obtained using a fluorescence microscope (Keyence, BZ-X700).

**Western blotting**

Proteins from the quadriceps and diaphragm were extracted using RIPA buffer (Nacalai tesque, 08714-04), in which 1% protease inhibitor cocktail (Nacalai tesque, 25955-11) was added. The samples were homogenized with a gentleMACS Dissociator (Miltenyi Biotec, USA) and further digested with sonication twice. After centrifugation at 150,000 rpm for 15 minutes, the supernatant was preserved at -80°C as a protein lysate. The concentration of protein lysate was measured using a BCA protein assay kit (Thermo Fisher Scientific, 23225) and EnVision 2104 multilabel plate reader (Perkin Elmer, USA) and plotted on the calibration curve. SDS-PAGE protein separation was performed with 4-12% Bis-Tris Plus gels (Invitrogen, NW04122BOX), and the proteins were transferred to a PVDF membrane (Invitrogen, IB401002) with an iBlot Gel Transfer Device (Thermo Fisher Scientific, USA). The protein amount aliquoted to each well was not equalized to induce maximum sensitivity for the COL6 detection. The membrane was blocked with Blocking one, and immunostaining was repeated as necessary. The first antibodies used were COL6 (Abcam, ab199720) and GAPDH (Millipore, #MAB374). For COL6 detection, the secondary antibody of biotinylated anti-rabbit IgG antibody (BA-1000, Vector Laboratories) and anti-streptavidin HRP conjugates (Biolegend, 405210) were used at dilutions of 1:500 and 1:20,000, respectively. For the detection of GAPDH, anti-mouse HRP conjugates (Invitrogen, #62-6520) were used at the dilution of 1:20,000. The membrane was incubated with ECL mixture (Super Signal^TM^ West Femto, Thermo Scientific, 34094) for 30 seconds and imaged with the ChemiDoc XRS+ Imaging System (Bio-RAD Laboratories, USA). The volume density of the COL6 band, which appeared at around 150 kDa, was analyzed with ImageLab^TM^ software (Bio-RAD Laboratories, USA). COL6 expression was shown relative to that in WT mice at 4 weeks after normalizing with the protein concentration measured in the BCA assay.

**Quantitative RT-PCR**

Tissues were well-minced with ophthalmic scissors and soaked in 1 mL sepazol (Nacalai tesque, 09379-84). Further homogenization was performed with a Biomasher II (Nippi, No.320102), and mRNA was extracted with isopropanol precipitation. 300 μg mRNA was reverse transcribed using the qPCR RT Master Mix as instructed (Toyobo, FSQ-301). cDNA mixed with SYBR Green PCR master mix (Thermo Fisher scientific, Ref 4367659) was duplicated for 40 cycles in quantitative PCR. The sequences of the primers are described in Supplemental Table 2.

Primary antibodies

| **Antigen** | **Catalogue No.** | **Host species** | **Dilution** |
| --- | --- | --- | --- |
| COL6 | Abcam, ab6588 | Rabbit | 1:200 |
| human-Lamin A/C | Leica, NCL-LAM-A/C | Mouse IgG2b | 1:200 |
| Laminin α2 | ALEXIS, ALX-804-190-C100 | Rat | 1:50 |
| MYH3 | Santa Cruz, sc-53091 | Mouse IgG1 | 1:50 |
| MYH3 | SIGMA, HPA021808 | Rabbit | 1:200 |
| MyoD | Santa Cruz, sc-760 | Rabbit | 1:500 |
| Human-nuclei | Millipore, MAB1281 | Mouse IgG1 | 1:200 |
| Pax7 | DSBH, AB528428 | Mouse IgG1 | 1:200 |
| Periostin | Abcam, ab14041 | Rabbit | 1:200 |
| human-PDGFRa | R&D, AF-307-NA | Goat | 1:20 |
| Single-stranded DNA | Sigma Aldrich, MAB3299 | Mouse | 1:50 |

Secondary antibodies

| **Species reactivity and conjugate** | **Host species** | **Catalog No.** | **Dilution** |
| --- | --- | --- | --- |
| Anti-goat | donkey | Thermo Fisher Scientific, A11057 | 1:500 |
| Anti-rabbit 488 | goat | Thermo Fisher Scientific, A11034 | 1:500 |
| Anti-rabbit 568 | goat | Thermo Fisher Scientific, A11036 | 1:500 |
| Anti-mouse IgG1 568 | goat | Thermo Fisher Scientific, A21124 | 1:500 |
| Anti-mouse IgG2b 568 | goat | Thermo Fisher Scientific, A21144 | 1:500 |
| Anti-rat 647 | goat | Thermo Fisher Scientific, A21247 | 1:500 |
| Anti-rabbit 488 | donkey | Thermo Fisher Scientific, R37118 | 1:500 |

**Supplementary Table 1.** Antibodies used for immunofluorescence and DAB staining.

|  | **Fw** | **Rv** |
| --- | --- | --- |
| ***Luciferase*** | GTGGTGTGCAGCGAGAATAG | CGCTCGTTGTAGATGTCGTTAG |
| ***m. Periostin*** | TAGCCCAATTAGGCTTGGCATCC | TAAGAAGGCGTTGGTCCATGCT |
| ***m. GAPDH*** | GGAGCGAGACCCCACTAACA | GCC TTCTCCATGGTGGTGAA |

h.: human m.: murine

**Supplementary Table 2.** Sequences of qPCR primers.
